# Combining single‐cell sequencing to identify key immune genes and construct the prognostic evaluation model for colon cancer patients

**DOI:** 10.1002/ctm2.465

**Published:** 2021-07-19

**Authors:** Jiasheng Xu, Siqi Dai, Kai Jiang, Qian Xiao, Ying Yuan, Kefeng Ding

**Affiliations:** ^1^ Department of Colorectal Surgery and Oncology Key Laboratory of Cancer Prevention and Intervention Ministry of Education The Second Affiliated Hospital Zhejiang University School of Medicine Hangzhou Zhejiang China; ^2^ Cancer Institute and Cancer Center, Zhejiang University Hangzhou Zhejiang China; ^3^ Department of Medical Oncology The Second Affiliated Hospital Zhejiang University School of Medicine Hangzhou Zhejiang China

Dear Editor,

Our immune gene model is of great help to predict the prognosis of colon cancer patients. The model genes could be used as prognostic markers and potential new targets for colon cancer patients.

Colon cancer is a common tumor with high incidence worldwide.^1^, ^2^ Recently, Immunotherapy has been found to be an effective anti‐cancer method, but it has not been used well in the treatment of colon cancer. Therefore, finding new prognostic‐related immune checkpoint genes in colon cancer is particularly important. In this study, we found the key prognostic immune genes of colon cancer and modeled them.

Different genes were analyzed by colon cancer data from TCGA database, and immune‐related genes were extracted. The immune differential genes were combined with the clinical data, and the model was constructed and evaluated. The model genes were enriched and analyzed. Single‐cell expression data of model genes were extracted, grouped according to the amount of each gene expression, and the percentage of infiltration of each cell phenotype in different groups was observed. The model genes were compared with immune checkpoints and immune cells. Analyzing the relationship between the key genes of the model and clinical information and using HPA database to verify model genes.

Three hundred ninety‐six immune differential genes were screened, and the corresponding heatmap and volcanic maps were plotted (Figures 1A and 1B). The results of enrichment analysis were showed in Table S2 and Figures 1C‐1F. PPI analysis illustrated that the interaction network between immune differential proteins was mainly enriched into seven interaction modules (Figures 1G and 1H). We obtained 187 immune differential genes (Table S3) that are related to survival. We identified 11 genes (the high expression of MET, NR5A2, TRGC2 was positively correlated with the prognosis of colon cancer patients,CD19, STC2, UCN, ULBP3, AEN, EBI3, TNFSF15, and high expression of CD3E was negatively correlated with prognosis in colon cancer patients) as key genes and modeled them by random forest algorithm (Figures 1I‐1S). This model was validated in randomly divided training sets (Figures 2A‐2C), validation sets (Figures 2F‐2H), and full sets (Figures 2K‐2M). The ROC curves of these three data showed AUC values were greater than 0.9 in 1, 3, and 5 years (Figures 2D, 2I, and 2N), and the survival rates of patients in the high and low risk group divided by this model were significantly different (Figures 2E, 2J, and 2O). The top five of the differential gene GO and KEGG analysis in the high risk group were shown in Figures 3A and 3B. The degree of immune infiltration of T cell subtypes was analyzed in the high and low expression groups of 11 key genes. The results were shown in Figures 3C‐3L. The expression of B, CD4T, CD8T, and Th1 cells in high and low risk groups in single cell colon cancer samples was significantly different (Figures 3M and 3N ). The model gene CD3E was highly positively correlated with LAG3, PDCD1, TIGIT immune checkpoint. TRGC2 was highly positive related to CD4, CD8T cells, and CD3E was highly positive related to CD4 and CD8 T cells (Figures 3O and 3P).

**FIGURE 1:**
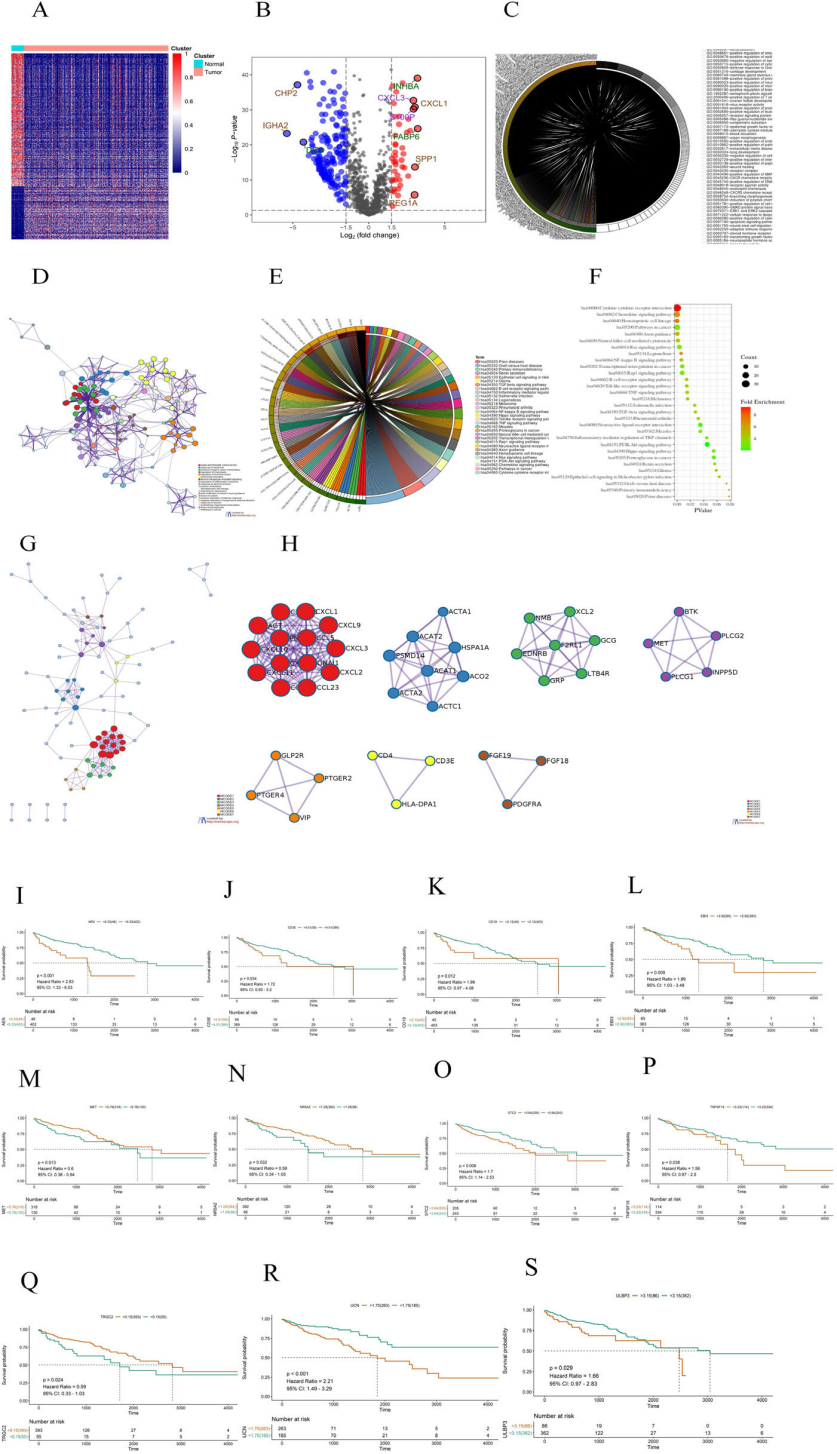
(A) Heatmap of immune differential genes. (B) Volcano map of immune differential genes. (C) Circle diagram drawn by GO analysis of immune differential genes. (D) GO analysis of the main biological processes involved in immune differential genes. (E) Circle diagram of KEGG enrichment analysis of immune differential gene. (F) Bubble diagram of KEGG enrichment analysis. (G) Protein interaction network diagram of immune differential genes. (H) Main seven modules in protein interaction network. (I‐S) Survival analysis results of 11 prognostic‐related immune differential genes

**FIGURE 2:**
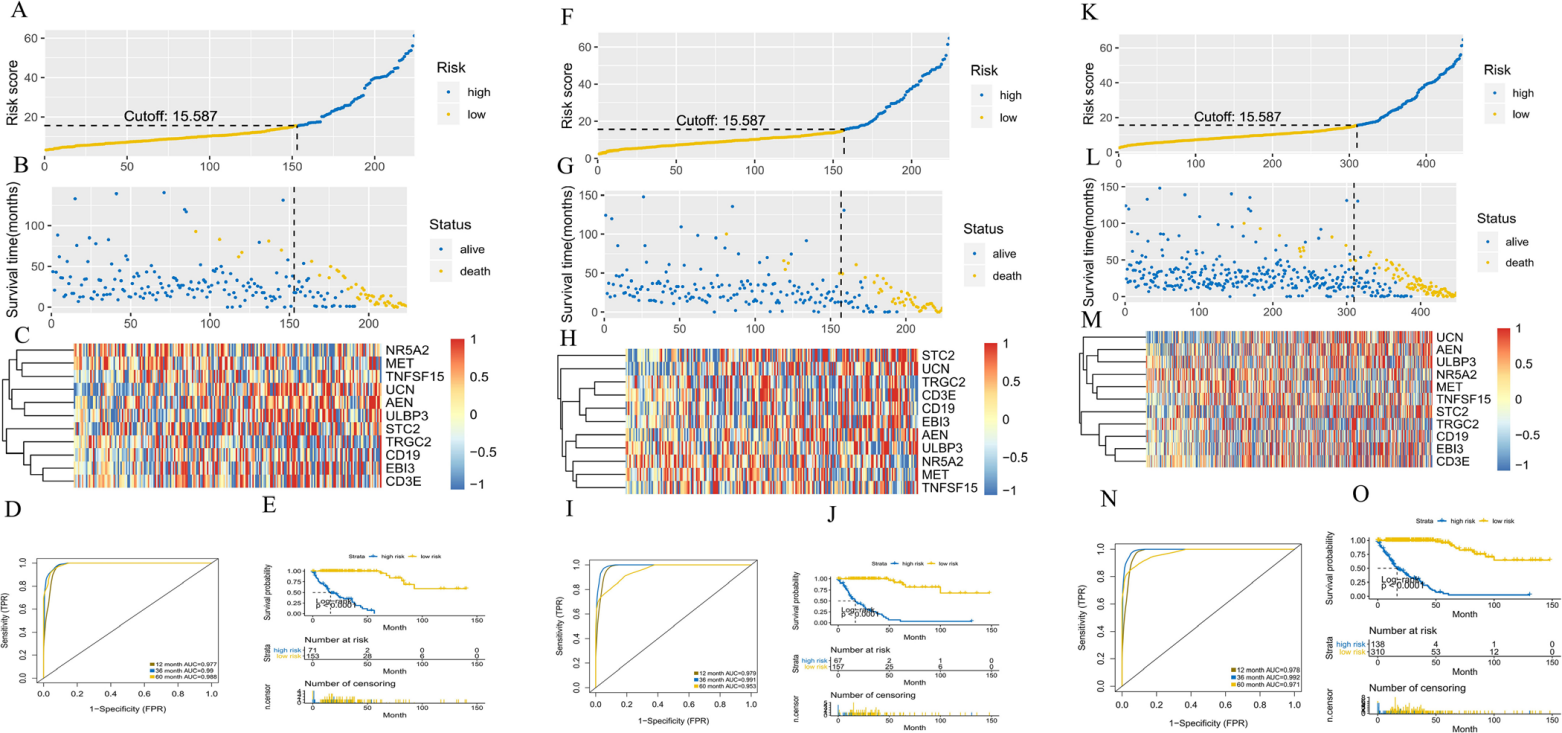
(A) The best split node for the training set to divide the high and low risk group. The abscissa is the number of patients in train group, the ordinate is the risk score value of the patient, and the high and low risk groups were classified by patient risk score divided by the risk score. (B) The abscissa is the number of patients in train group, and the division of high risk and low‐risk groups is verified by survival. (C) Heatmap of the expression of 11 key prognostic genes in high‐risk and low‐risk patients of train group. (D) ROC analysis test results of sensitivity and specificity of the train model. (E) Comparison of survival analysis between high‐risk and low‐risk patients of train group. (F) The best split node for the test set to divide the high‐risk group. The abscissa is the number of patients in test group, the ordinate is the risk score value of the patient, and the high and low risk groups are divided by the risk score. (G) The abscissa is the number of patients in test group, and the division of high risk and low‐risk groups is verified by survival. (H) Heatmap of the expression of 11 key prognostic genes in high‐risk and low‐risk patients of test group. (I) ROC analysis test results of sensitivity and specificity of the test model. (J) Comparison of survival analysis between high‐risk and low‐risk patients of test group. (K) The best split node for the total set to divide the high‐risk group. The abscissa is the number of patients in total group, the ordinate is the risk score value of the patient, and the high and low risk groups are divided by the risk score. (L) The abscissa is the number of patients in total group, and the division of high risk and low‐risk groups is verified by survival. (M) Heatmap of the expression of 11 key prognostic genes in high‐risk and low‐risk patients of total group. (N) ROC analysis test results of sensitivity and specificity of the total model. (O) Comparison of survival analysis between high‐risk and low‐risk patients of total group

**FIGURE 3:**
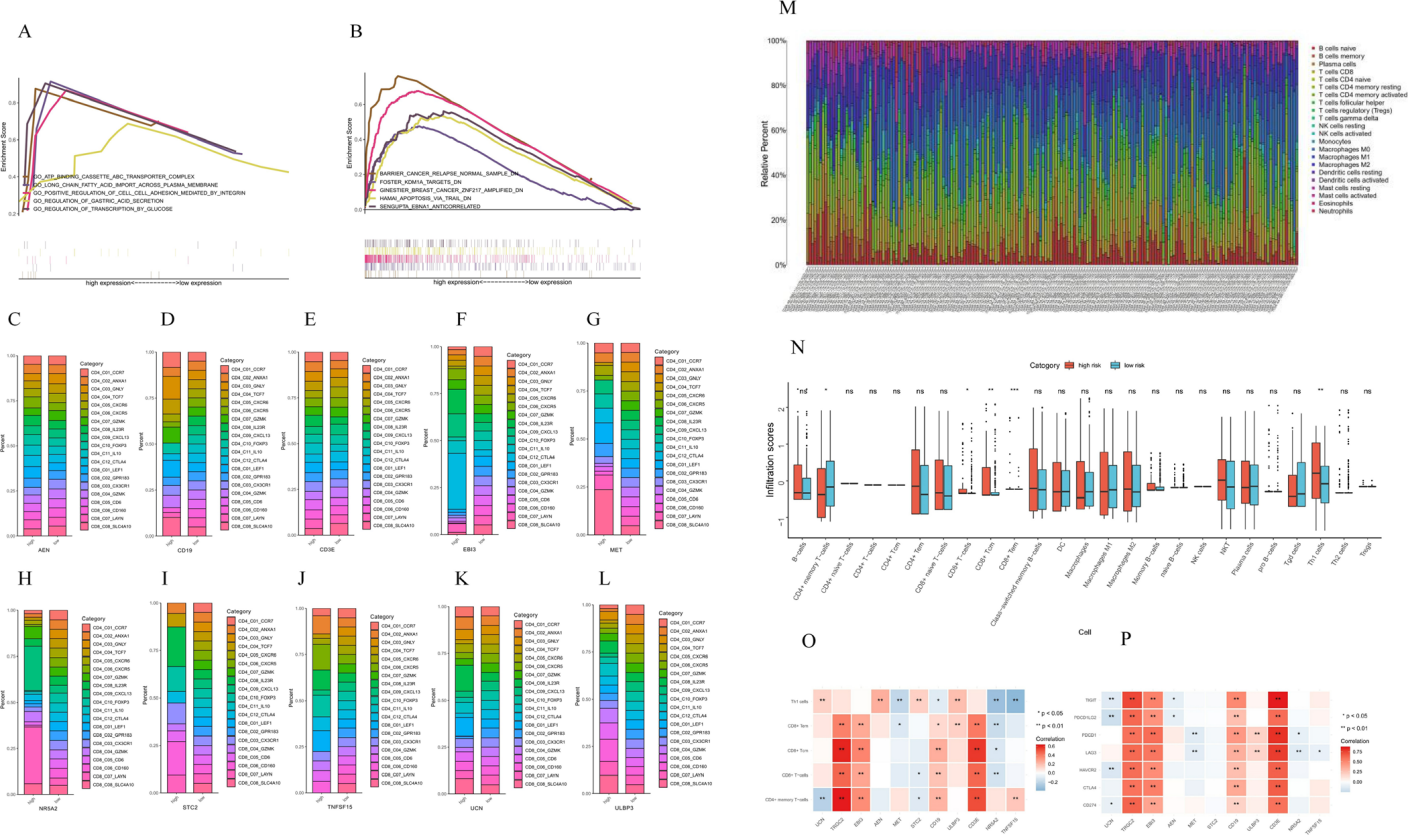
(A) GO enrichment analysis results of differential genes in the high and low risk groups. (B) KEGG enrichment analysis results of the differential genes in the high and low risk groups. (C‐L) Based on single cell data, divided by 10 hub genes except TRGC2. The expression of each T cell subtype in the high and low expression group. (M) The infiltration of 22 kinds of immune cells in the tumor tissue of the patient. (N) Infiltration of 25 kinds of immune cells in the high‐ and low‐risk groups. (O) Correlation analysis results between model gene expression and differentially expressed immune cells. (P) Correlation analysis results between model genes expression and immune checkpoints

The CD19 expression of tumor tissue was related to age (Figure S1A). The degree of expression of CD3E, EBI3, STC2, and TRGC2 genes was associated with the anatomical location of the tumor (Figure S1B). The expression of EBI3 and UCN genes was related to mismatch repair (Figure S1C). UCN gene expression was associated with the occurrence of lymphatic invasion (Figure S1D). The expression of AEN, CD3E, TRGC2, and ULBP3 was different when microsatellite was highly unstable (MSIH) and at MSS (Figure S1E). The expression of STC2 and TNFSF15 genes was positively correlated with tumor in situ invasion (Figure S1F). The expression of AEN, STC2, TRGC2, and ULBP3 genes was related to the degree of lymphatic metastasis (Figure S1G). The expression of CD3E, EBI3, and TRGC2 genes was related to the degree of distant metastasis (Figure S1H). The expression of CD3E, STC2, and TRGC2 genes was related to the clinical stage of patients (Figure S1I). The expression of CD3E gene was associated with the recurrence of tumor after treatment (Figure S1J). The expression of UCN genes was closely related to the effect of initial treatment (Figure S1K). The validation results were consistent with the results of the study analysis (Figure S1L).

After analysis of single‐cell samples, we found that IL‐23R expression increased in the EBI3, NR5A2, STC2, and UCN high expression group. Studies have shown that targeting IL‐23 inhibits the growth of inflammation‐related cancers.^3^ As a result, these four genes may influence colon cancer growth by influencing IL‐23R. CD4T and Th1 cells are inextricably linked to the development of colon cancer, which has been proved to promote the precancerous lesions of colon cancer‐the pathogenesis of inflammatory bowel disease.^4^ The inhibition of TIGIT enhances tumor‐specific T cellular immunity, so can enhance PD‐1 ligand PD‐L1 antibody therapy, thus enhancing the sustained memory immune function of tumor reactivation.^5^ LAG3 high expression indicates better prognostic in colon cancer patients.^6^ Besides, we found a high positive correlation between TRGC2, CD3E and TIGIT, PDCD1, LAG3. This means that TRGC2 and CD3E can influence the growth of colon cancer not only by affecting the expression of immune cells but also by acting on TIGIT, PDCD1, and LAG3 immune checkpoints to affect the prognosis of patients.

In conclusion, combined with the data of single cell sequencing, our study identified 11 immune‐related genes significantly associated with the prognosis of colon cancer patients and constructed the prognostic evaluation model. The model was verified by multiple data sets to prove that it was accurate and reliable.

## Supporting information

Supporting information

Supporting information

Supporting information

Supporting information

Supporting information

Supporting information.
Figure S1 (A) Correlation analysis between the expression of model gene CD19 and patient age. (B) Correlation analysis between model gene expression and tumor anatomical location. (C) Correlation analysis between model gene expression and occurrence of mismatch repair. (D ) Correlation analysis between the expression of model gene UCN and the occurrence of lymphatic invasion. (E) Correlation analysis between the expression of model gene and the occurrence of microsatellite instability. (F) Correlation analysis between model gene expression and tumor TNM staging. (G) Correlation analysis between model gene expression and lymphatic metastasis. (H) Correlation analysis between model gene expression and distant metastasis. (I) Correlation analysis between model gene expression and clinical stage of patients. (J) Correlation analysis between CD3E gene expression and tumor recurrence after treatment. (K) Correlation analysis between UCN gene expression and initial treatment effect. (L) The expression of prognostic‐related immune differential genes in colon cancer tumor tissues and normal tissues
