# Supplementary material for: Combining single‐cell sequencing to identify key immune genes and construct the prognostic evaluation model for colon cancer patients
Source: Clin Transl Med. 2021 Jul 19;11(7):e465. doi: 10.1002/ctm2.465 (PMC8288000; doi:10.1002/ctm2.465)
Supplement: Supplementary file 1 — Supporting information [file CTM2-11-e465-s004.doc]

**2 Methods**

2.1 Data download, processing and access to immune-related genes

The 514 data of COAD patients were downloaded from the TCGA database to remove duplicate and low expression genes; The data was transferred from FPKM format to TPM form to complete data quality control. Downloading immune genes from immport website and extracting immune gene data from quality control data.

2.2Screening and analysis of immune differential genes

Normalizing of data of immune-related genes extracted from the gene expression data of adjacent and cancerous tissues, differences analysis of the normalized data by limma[15] package was used to obtain immune differential genes.The obtained immune differential genes were visualized to obtain thermal and volcanic maps of immune differential genes. We used the "clusterProfiler"[PMID：22455463] function package in R language for GO (including Biological Process、Molecular Function and Cellular Component) and KEGG Pathway enrichment analysis for the resulting immune differential genes. At P value <0.05 we considered the corresponding entries to be significantly enriched. Metascape database is an online website that analyzes and predicts the functional connections and interactions of proteins. We used Metascape (https://metascape.org/gp/index.html #/ main/step1) for protein network interaction analysis （protein–protein interaction (PPI) networks, the functional and protein interactions of the proteins corresponding to the immune differential genes were analyzed, and the interaction modules of these proteins were further analyzed.

2.3 Extraction of clinical data and modelling

Survival analysis by X-tile[16] software to obtain the best cut-off points for survival analysis of each immune-related gene. Extract the survival matrix of the genes that affect the clinical prognosis; carry on the random forest training study, after the training study, divide the patients into high risk group and low risk group, and get the hub gene at the same time. The patients were randomly divided into training set and validation set.The training set was trained bythe random forest which trained 1000 cycles through the minimum depth method to obtain the best results and construct the model. Finally, the training results were verified in the validation set and the full set (including the training set and validation set).

2.4 GSEA enrichment analysis

The high and low risk groups divided according to the training results of random forest model were analyzed by GSEA software respectively. The results were sorted according to the P values from small to large, and the top five pathways with the lowest P values were selected to draw multiple GSEA maps.

2.5 Single cell data download and model gene expression extraction for colon cancer

Download single cell data chips associated with colon cancer from GEO GSE108989, and extract hub genes from the model. Through analyzing the expression of each hub gene in the single cell data sample, the single cell sample was divided into high and low expression groups with the median expression amount, and the relative infiltration abundance of CD4,CD8 subpopulation cells in the high and low expression group of each hub gene was observed and compared respectively.

2.6 Analysis of immune cell infiltration and correlation between model genes and immune cells in colorectal cancer data

Calculate the infiltration abundance of immune cells in the COAD, and merge the cell abundance with risk group; draw the difference map of cell infiltration in the high and low risk group. Combine gene expression matrix and cell infiltration fraction matrix; draw correlation heat map pictures to show the correlation between differential cells and hub genes.

The heat map between hubgene and immune checkpoint was drawn based on immune checkpoint expression matrix

2.7 Correlation between model genes and clinical indicators

Model genes were divided into optimal intercept points and survival analysis by X-tile software.Correlation analysis was conducted between model genes and clinical indicators to explore the relationship between model genes and 14 clinical indicators, such as patient age, sex, TNM stage, tumor anatomical location, mismatch repair, microsatellite instability, polyp, clinical stage, tumor recurrence, and therapeutic effect.

2.8 Prognosis-related gene expression validation

Validate gene expression of selected prognostic-related autophagy genes. A HPA database was used to verify the expression of prognostic-related autophagy genes in colon cancer tumor tissues and normal tissues, and to compare whether the expression difference was consistent with the previous analysis.

2.9 Statistical analysis

Kaplan-Meier method was used to estimate the overall survival rate of different groups, and log-rank was used to test the significance of the difference between different groups. Utilize the wilcoxon sign rank sum test to compare the infiltration differences of immune cells in different groups, with p <0.05 as the significant threshold.R software was used for statistical analysis, and the version number was V3.5.2.
