# Supplementary material for: Combining single‐cell sequencing to identify key immune genes and construct the prognostic evaluation model for colon cancer patients
Source: Clin Transl Med. 2021 Jul 19;11(7):e465. doi: 10.1002/ctm2.465 (PMC8288000; doi:10.1002/ctm2.465)
Supplement: Supplementary file 2 — Supporting information [file CTM2-11-e465-s005.pdf]

Random Forests for Survival, Regression, and Classification

A Parallel Package for a General Implemention of Breiman's Random Forests

Theory and Specifications

Udaya Kogalur & Hemant Ishwaran

1. 1. Table of Contents

1. Table of Contents
2. Introduction
3. Package Overview
    3.1. Splitting and Node Size
    3.2. Model Overview
4. Variable Selection
    4.1. Minimal Depth and Maximal Subtrees
    4.2. Variable Importance
5. Imputation
6. Prediction
    6.1. outcome = "test"
    6.2. Pruning
7. Hybrid Parallel Processing
8. Theory and Specifications
    8.1. Survival
    8.2. Competing Risk
    8.3. Regression
    8.4. Classification
    8.5. Multivariate
    8.6. Unsupervised
9. Supplementary Code
10. References

1. 1. Table of Contents
2. 2. Introduction
3. 3. Package Overview
    3.1. 3.1. Splitting and Node Size
    3.2. 3.2. Model Overview
4. 4. Variable Selection
    4.1. 4.1. Minimal Depth and Maximal Subtrees
    4.2. 4.2. Variable Importance
5. 5. Imputation
6. 6. Prediction
    6.1. 6.1. outcome = "test"
    6.2. 6.2. Pruning
7. 7. Hybrid Parallel Processing
8. 8. Theory and Specifications
    8.1. 8.1. Survival
    8.2. 8.2. Competing Risk
    8.3. 8.3. Regression
    8.4. 8.4. Classification
    8.5. 8.5. Multivariate
    8.6. 8.6. Unsupervised
9. 9. Supplementary Code
10. 10. References

2. 2. Introduction

Random Forests for Survival, Regression, and Classification (RF-SRC) is an ensemble tree method for the analysis of data sets using a variety of models. As is well known, constructing ensembles from base learners such as trees can significantly improve learning performance. It was shown by [Breiman, 2001] that ensemble learning can be further improved by injecting randomization into the base learning process --- a method called Random Forests. RF-SRC extends Breiman's Random Forests method and provides a unified treatment of the methodology for models including right censored survival (single and multiple event competing risk), multivariate regression or classification, and mixed outcome (more than one continuous, discrete, and/or categorical outcome). When one continuous or categorical outcome is present, the model reduces to univariate regression or classification respectively. When no outcome is present, the model implements unsupervised learning. The [Five Models] figure summarizes the situation.

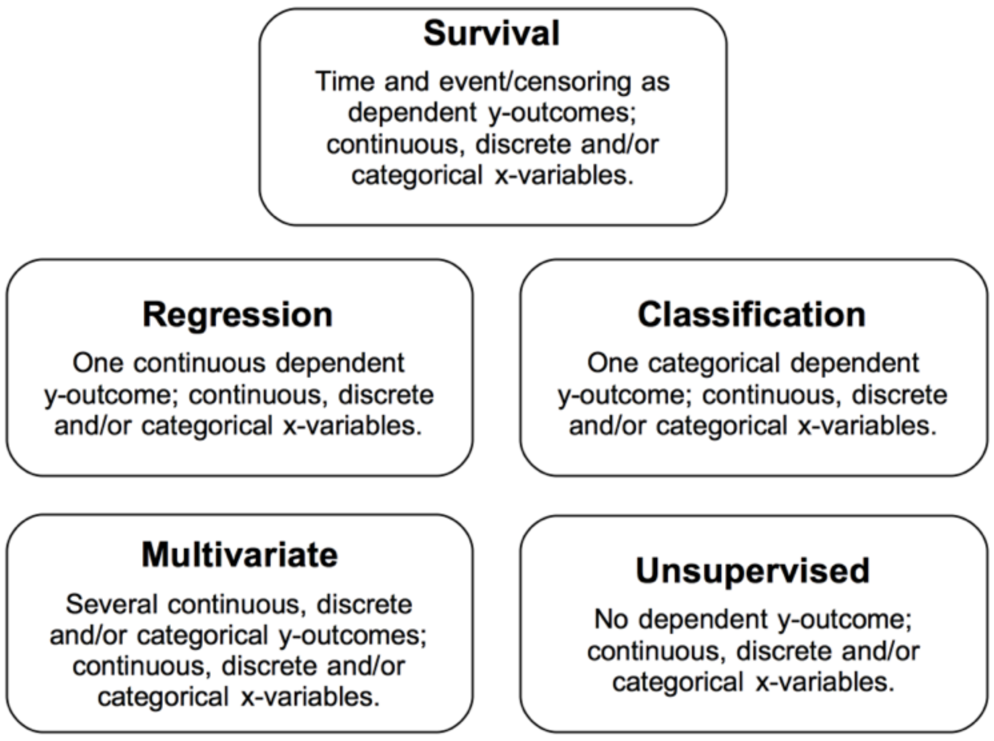

Five Models Implemented in RF-SRC

RF-SRC introduces new split rules for each model and gives the user the ability to define and code custom split rules. Deterministic or random splitting is available for all models. Variable predictiveness can be assessed using variable importance measures [Ishwaran et al., 2007] for single as well as grouped variables. Variable selection is implemented using minimal depth [Ishwaran et al., 2010]. Missing data (for x-variables and y-outcomes) can be imputed on both training and test data. The package supports shared memory parallel processing on desktop computers via OpenMP. The package also supports hybrid parallel processing via OpenMP & Open MPI on clusters that implement distributed memory. Execution time is reduced almost linearly with respect to the cores available.

An overview of this document follows: In the [package overview] we describe the Random Forests algorithm highlighting the recursivity that is at its core. The formulas, split rules, terminal node estimators, and prediction errors used in the five models are described briefly. In the section on [Variable Selection] the primary approaches for variable predictiveness, variable selection and identifying interactions between variables are discussed. In the section on [Imputation] the approach to handle missingness is discussed. In the section on [Prediction] a brief discussion of training, restoration, test data, and pruning is given. In the section on [Hybrid Parallel Processing] we demonstrate that the package is capable of hybrid parallel computation in a non-trivial and massive way, and provide details of how this can be achieved. In the [Theory and Specifications] section we provide the mathematics for the split rules, terminal node estimators, and prediction error for each family.

3. 3. Package Overview

Building a Random Forests model involves growing a binary tree [Breiman, 2001] using user supplied training data and parameters. As is shown in the [Five Models] figure, data types must be real valued, discrete or categorical. The response can be right-censored time and censoring information, or any combination of real, discrete or categorical information. The response can also be absent entirely. The resulting forest contains many useful values which can be directly extracted by the user and parsed using additional functions. The basic algorithm for growing a forest is provided in this [Recursive Algorithm]. The process iterates over `ntree`, the number of trees that are to be grown. In practice, the iteration is actually parallelized and trees are grown concurrently, not iteratively. This is explained in more detail in the section on [Hybrid Parallel Processing]. The recursive nature of the algorithm is reflected in the repeated calls to split a node until conditions determine that a node is terminal. Another key aspect of the algorithm is the injection of randomization during model creation. Randomization reduces variation. Bootstrapping [Breiman, 1996] at the root node reduces variation. Feature selection is also randomized with the use of the parameter `mtry`. In the [Recursive Algorithm] `N` is defined as the number of records in the data set, and `P` as the number of x-variables in the data set. The parameter `mtry` is such that `1 <= mtry <= P`. At each node, the algorithm selects `mtry` random x-variables according to a probability vector `xvar.wt`. The resulting subset of x-variables are examined for best splits according to a `splitrule`. The parameter `nsplit` also allows one to specify the number of random split points at which an x-variable is tested. The depth of trees can be controlled using the parameters `nodesize` and `nodedepth`. The parameter `nodesize` ensures that the average nodesize across the forest will be at least `nodesize`. The parameter `nodedepth` forces the termination of splitting when the depth of a node reaches the value specified. Node depth is zero-based from the root node onward. Reasonable models can be formed with the judicious selection of `mtry`, `nsplit`, `nodesize`, and `nodedepth` without exhaustive and deterministic splitting.

```
// The result of this algorithm is a forest of binary trees, each of
// which partitions the covariate space into hyper-cubes, yields
// ensemble statistics for each individual based on terminal node
// membership across the forest, and allows model recovery for
// prediction, proximity, importance and much more.
function GROW(
        data$, // Data containing y-outcomes and x-variables.
        $N$, // Number of records in data.
```

```
        $P$, // Number of x-variables in data.
        $ntree$, // Number of trees in the forest.
        $splitrule$, // Split rule and formula.
        $mtry$, // Size of the random subset of $\{1,...,P\}$.
        $xvar.wt$, // Probabilities of selecting an x-var as an $mtry$ candidate.
        $nodesize$, // Average node size over the forest.
        $nodedepth$, // Maximum node depth.
        $nsplit$, // Size of random split points for each $mtry$ candidate.
      )
for ($i = 1$ to $ntree$)
{
  Bootstrap the $data$, creating an in-bag, and an out-of-bag
  subset. Make the root node, and tag all individuals as
  members of this node.
  recurse until node fails to split
  {
    if ((size of node $\ge 2 \times nodesize$) and
    (depth of node $\ge nodedepth$) and (node is impure))
    {
      Select $mtry$ covariates according to weight vector $xvar.wt$.
      for each $mtry$ covariate
      {
        Conduct deterministic or random splitting according to $nsplit$.
        {
          Use the specified splitrule to determine the
          split statistic. Save the covariate and split
          point when the split statistic is better than
          those proceeding it.
        }
      }
      if a best split exists
      {
        Split the node at the best split point into a left
        and right daughter. Tag all individuals according
        to their daughter membership.
      }
    }
    if the node was split
    {
      recurse using the left daughter
      recurse using the right daughter
    }
  }
  Save terminal node information.
  for each out-of-bag individual
  {
    Update the ensemble using the terminal node statistics.
  }
  Update performance measures, proximity and importance statistics.
}
Normalize out-of-bag ensemble outputs. Save forest topology, and
terminal node membership to allow prediction.

// The result of this algorithm is a forest of binary trees, each of
// which partitions the covariate space into hyper-cubes, yields
// ensemble statistics for each individual based on terminal node
// membership across the forest, and allows model recovery for
// prediction, proximity, importance and much more.
function GROW(
        data$, // Data containing y-outcomes and x-variables.
        $N$, // Number of records in data.
```

```
        $P$, // Number of x-variables in data.
        $ntree$, // Number of trees in the forest.
        $splitrule$, // Split rule and formula.
        $mtry$, // Size of the random subset of $\{1,...,P\}$.
        $xvar.wt$, // Probabilities of selecting an x-var as an $mtry$ candidate.
        $nodesize$, // Average node size over the forest.
        $nodedepth$, // Maximum node depth.
        $nsplit$, // Size of random split points for each $mtry$ candidate.
      )
for ($i = 1$ to $ntree$)
```

```
{
   Bootstrap the $data$, creating an in-bag, and an out-of-bag
   subset. Make the root node, and tag all individuals as
   members of this node.
   recurse until node fails to split
   {
      if ((size of node $\ge 2 \times nodesize$) and
      (depth of node $\ge nodedepth$) and (node is impure))
      {
         Select $mtry$ covariates according to weight vector $xvar.wt$.
         for each $mtry$ covariate
         {
            Conduct deterministic or random splitting according to $nsplit$.
            {
               Use the specified splitrule to determine the
               split statistic. Save the covariate and split
               point when the split statistic is better than
               those proceeding it.
            }
         }
         if a best split exists
         {
            Split the node at the best split point into a left
            and right daughter. Tag all individuals according
            to their daughter membership.
         }
      }
      if the node was split
      {
         recurse using the left daughter
         recurse using the right daughter
      }
   }
   Save terminal node information.
   for each out-of-bag individual
   {
      Update the ensemble using the terminal node statistics.
   }
   Update performance measures, proximity and importance statistics.
}
Normalize out-of-bag ensemble outputs. Save forest topology, and
terminal node membership to allow prediction.
```

Recursive Grow Algorithm

A simulated classification training data set is shown on the left of the figure labeled [Tree Decision Boundary]. The data set has two real valued features, x_1 and x_2. The response is a class label that can take on four values. The class sizes are equal. Each class covers a circular area. The circles touch at a single point – the origin. At this point, four data points with all four class labels exist. On the right of the figure is a tree produced from the training data with associated split points labeled. The tree was grown using bootstrapping [Breiman, 1996]. Bootstrapping results in well-defined subsets. The bootstrap, some of which are duplicates, are members of the in-bag (IB) subset. The remaining individuals define the out-of-bag (OOB) subset for the tree. See [Breiman, 1996] for a detailed description of these concepts. Only the bootstrap is used to train the model and to define terminal node statistics. In this model, the terminal node statistic is the resulting frequency distribution of the class labels in a terminal node. This distribution is shown under each terminal node in the right of the figure. The decision boundary on x_1 and x_2 formed by the tree is superimposed on the data space along with the six terminal node labels.

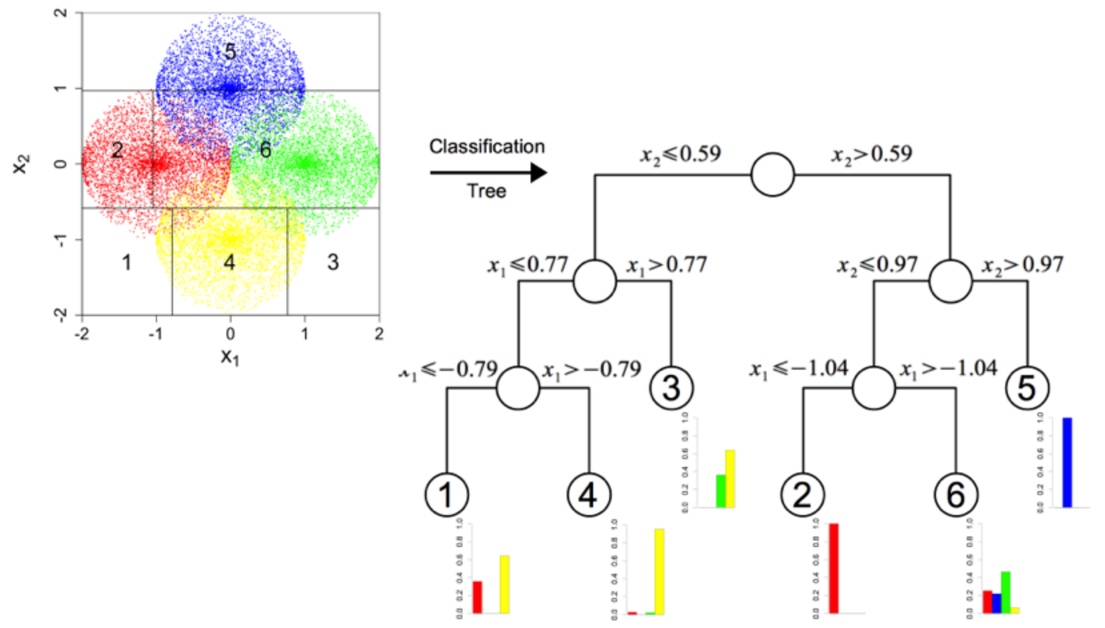

Tree Decision Boundary

The OOB subset for a tree does not play a role in determining its topology. Each individual in the OOB subset for a tree is passive and assigned a unique terminal node membership and terminal node statistic. An OOB ensemble statistic for each individual is formed by combining the terminal node statistics from all trees in which an individual is an OOB member. The class with the maximum frequency in the OOB ensemble statistic serves as the predicted class label for the member. In the figure labeled [Forest Decision Boundary] a single test data point centered at the origin is sent into the previously grown forest for prediction. Each tree provides a unique terminal node membership and statistic for this test individual. The resulting forest ensemble statistic yields a class with the maximum frequency as the predicted class label for this test data point. We note that the probability of all classes are roughly equal. This is because the training data set has one case in each class at the origin. The maximum class frequency for this example is yellow, though we can ascribe this to Monte Carlo effects. When the x-variable space is densely covered with test data points, the predicted values for the data space reveal the forest decision boundary. The boundary is also shown in the lower half of the figure.

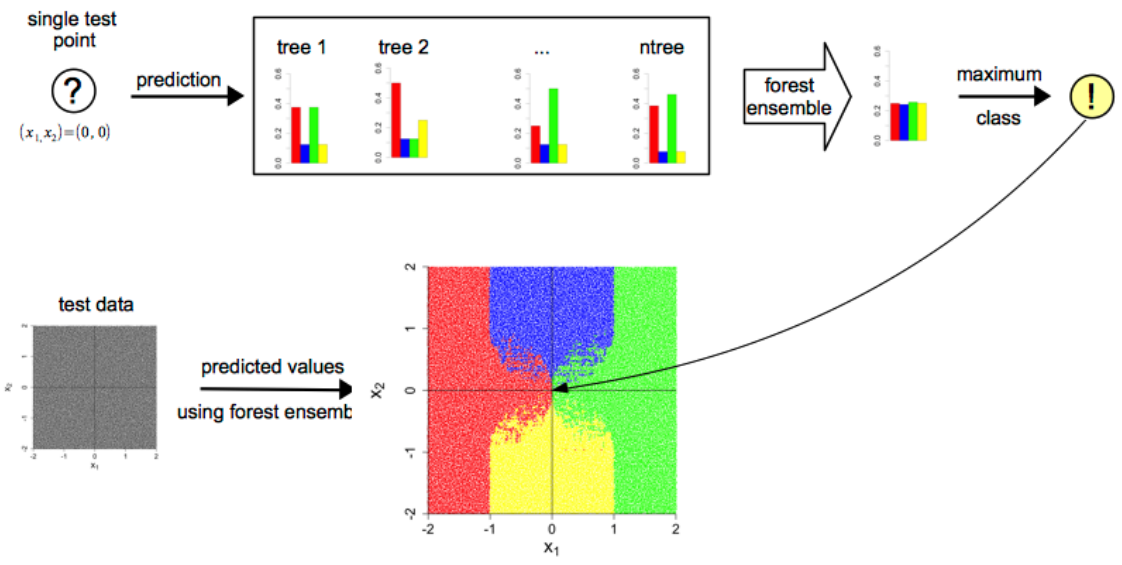

Forest Decision Boundary

3.1. 3.1. Splitting and Node Size

It is important to understand how splitting and node size affect the topology of a tree. To aid in this, some notation is introduced first. Assume h is the node of a tree, and that the task is to split h into two daughter nodes. Assume there are `n` cases in the node h. Some of these may be replicates if bootstrapping is in effect. At the root node, `n = N`. Let there be `P` x-variables in the data set. These can be real valued, discrete, or categorical. Let $\mathbf{X}_i$ be the `P`-dimensional feature for a case i such that $\mathbf{X}_i = (X_{i1}, \dots, X_{i\mathrm{P}})$. Let x be the observed value for the pth feature in the data set, where $p \in \{1, \dots, \mathrm{P}\}$.

Real versus categorical splitting

If x is continuous or discrete, a proposed split of h on x is always of the form $x \leq c$ and $x > c$. Such a split defines left and right daughter membership according to an individual's value X_{ip}. A deterministic split on x requires considering all unique values of x in the node h.

If x is categorical, denoting the split is more complex. For computational coherence, categorical x-variables (and y-outcomes for that matter) are treated as factors with an immutable 1:1 mapping of categories to integers. Thus, an x-variable with f categories (or levels) is uniquely mapped to f integers $\{1, \dots, f\}$. A split on a factor with f levels in the node h requires dividing the levels into two complementary subsets. Left and right daughter membership is determined according to an individual's level X_{ip} and to which of the two complementary subsets it belongs. A deterministic split on a factor requires considering all possible complementary pairing of levels. For example, a factor with six levels in a node has

$$C(6, 1) + C(6, 2) + C(6, 3)$$

possible splits. These possibilities can be considered to be composed of three groups. The first group has one level going left, and five levels going right. There are $C(6, 1)$ such splits. The second group has two levels going left and four levels going right. There are $C(6, 2)$ such splits. The third group has three levels going left and three levels going right. There are $C(6, 3)$ such splits. These three groups comprise the cardinal group count. In general, there are $floor(f/2)$ cardinal groups. The total number of possible splits (complementary pairings) is $2^{f-1} - 1$.

Deterministic splitting on factors becomes problematic because of the large number of permutations as the number of levels rise. There is a practical limit on the number of levels a factor can have, within our implementation. It is imposed by `UNIT_MAX` in `limits.h` on the operating system in question. A typical value for this is $2^{32} - 1$ on most machines. Overrides are in place to avoid deterministic splitting on large factors. The nature of the override is dependent on whether the user has specified `splitrule = "random"` and/or `nsplit > 0`. In general, we try to limit the number of split attempts to be less than the node size. By definition, this is always case when considering continuous x-variables. That is, if a node has n' bootstrap cases, there will be at most n' values on which to split. To encourage good splits, the algorithm attempts to emulate the same action on factors. In some cases this will result in deterministic splitting. In others, it will involve sampling from the $2^{f-1} - 1$ complementary pairings. Whether factor or continuous x-variable, the sampling is never greater than the nodesize regardless of what value of `nsplit` has been set.

Formulas for splits on continuous x-variables are more easily conveyed to the reader because left and right daughter splits depend on a simple inequality. For this reason, the split rules described in the [Theory and Specifications] section on discrete and continuous x-variables. However, the methodology is exactly applicable to factor x-variables. In addition, for clarity the formulas described are for splits at the root node, where $n = N$.

Deterministic versus random splitting

In contrast to deterministic splitting, a random version of any split rule may be invoked using the parameter `nsplit`. When `nsplit = 0`, deterministic splitting occurs and all possible split points for each of the `mtry` variables are considered. If `nsplit` is set to a non-zero positive integer, then a maximum of `nsplit` split points are randomly chosen for each of the `mtry` variables within a node. The split rule is applied to the randomly chosen split points, and the node is split on that x-variable and split value yielding the best split statistic as measured by the split rule. Pure random splitting can be invoked by setting `splitrule="random"`. In this case, for each node, a variable is randomly selected and the node is split using one randomly chosen split point. See [Cutler and Zhao, 2001] and [Lin and Jeon, 2006] for more details.

Trees tend to favor splits on continuous variables [Loh and Shih, 1997], so it is good practice to use the `nsplit` option when the data contains a mix of continuous and discrete variables. Using a reasonably small value mitigates bias and may not compromise accuracy. The value of `nsplit` has a significant impact on the time taken to grow a forest. When non-random splitting is in effect, i.e. `nsplit = 0`, iterating over each split point can be CPU intensive. However, when `nsplit > 0`, or when pure random splitting is in effect, CPU times are drastically reduced.

Node depth and node size

Node size and node depth are the most explicit means of controlling the growth of a tree. The parameter `nodedepth` is defined as the number of connections between the node and the root node. It is zero-based, thus defining the root node as having depth zero. Setting `nodedepth` limits the maximum depth to which a tree can be grown. The default behaviour is to ignore this parameter and not limit the depth of a tree. In contrast, `nodesize` is always respected and is crucial to achieving a good model. When `nodesize` is set too large, the forest will be under-grow and model accuracy will be compromised. When it is set too small, the potential to split on noisy x-variables increases, the forest is over-grown and model accuracy can actually deteriorate past a certain threshold value for `nodesize`. We define `nodesize` as the average number of bootstrap cases that will be found in a terminal node in a tree in the forest. Splitting can terminate well before this condition is reached if node purity is detected before attempting to split a node. From the [Recursive Algorithm] it can be seen that there are three conditions necessary for splitting a node:

1. The current node depth must be less than the maximum node depth allowed.
2. The current node size must be at least 2 times the node size specified.
3. The current node must be impure.

Condition (2) assures us that if a split occurs on a node with 2 times the node size specified, the average node size of the pair of daughters will be `nodesize`. If one daughter is less than `nodesize`, the other daughter will be greater than `nodesize`. But such terminal nodes will average to `nodesize` over the forest.

3.2. 3.2. Model Overview

This section is intended as an overview to orient the reader to more high-level package details concerning the [Five Models] produced by the package. Each model requires a slightly different formula specification, and uses model-specific split rules. This results in model-specific terminal node statistics, ensembles, and a model-specific prediction error algorithm. For completeness and rigour, the mathematical details of all models are given in the [Theory and Specifications] section. Survival, Competing Risk, Regression, Classification, Multivariate and Unsupervised split rules are described, along with the terminal node estimators, and the prediction error for these families. In addition, a short code example for each model is given.

In the [Formula Specification] table, example grow calls for the five models are listed. The data sets used in the two Survival function calls are available in `randomForestSRC`. The data sets used in the remaining function calls are available from the base R installation. Survival settings require a time and censoring variable which need to be identified in the formula as the response using the standard `Surv` formula specification. Regression and Classification settings emulate the formula specifications of the `randomForest` package. In the Multivariate and Unsupervised case, there are variants for the formula, depending on how many y-outcomes are to be specified as the response. These variants are given in more detail in this section.

Family	Example Grow Call with Formula Specification
Survival	`rfsrc(Surv(time, status) ~ ., data=veteran)`
Competing Risk	`rfsrc(Surv(time, status) ~ ., data=wihs)`
Regression	`rfsrc(Ozone ~., data=airquality)`
Classification	`rfsrc(Species ~., data=iris)`
Multivariate	`rfsrc(Multivar(mpg, cyl) ~., data=mtcars)`
Unsupervised	`rfsrc(Unsupervised() ~., data=mtcars)`

In the [Split Rules] table, the rule in *italics* denotes the default split rule for each model. The default split rule is applied when the user does not specify a split rule. The package uses the data set and formula specification to determine the model. Survival and Competing Risk both have two split rules. Regression has three flavours of split rules based on mean-squared error. Classification also has three flavours of split rules based on the Gini index. The Multivariate and Unsupervised split rules are a composite rule based on Regression and Classification. Each component of the composite is normalized so that the magnitude of any one y-outcome does not influence the statistic. See the [Theory and Specifications] section for more details on the mathematics of the split for each family.

Family	Split Rules
Survival	*log-rank* (1)
	log-rank score (2)
Competing Risk	*log-rank modified weighted* (12)
	log-rank (11)
Regression	*mean-squared error weighted* (16)
	mean-squared error unweighted (17)
	mean-squared error heavy weighted (18)
Classification	*Gini index weighted* (22)
	Gini index unweighted (23)
	Gini index heavy weighted (24)
Multivariate	Composite mean-squared error
	Composite Gini index
Unsupervised	Composite mean-squared error
	Composite Gini index

All models also allow the user to define a custom split rule statistic. Some basic C-programming skills are required. Examples for all the families reside in the C source code directory of the package in the file `splitCustom.c`. Note that recompiling and re-installing the package is necessary after modifying the source code.

In the table [Terminal Node Statistics] (TNS), the terminal node estimators produced by the five models are summarized. For Survival, the TNS is the Kaplan-Meier estimator at the time points of interest specified by the user, or as determined by the package if not specified. Competing Risk has two TNS's: the cause-specific cumulative hazard estimate, and the cause-specific cumulative incidence function. Regression and Classification TNS's are the mean and class proportions respectively. For a Multivariate model, there are TNS's for each response, whether it is real valued, discrete or categorical. The Unsupervised model has no TNS, as the analysis is responseless. See the [Theory and Specifications] section for more details on the mathematics of the TNS for each family.

Family	Terminal Node Statistics
Survival	Kaplan-Meier (3)
Competing Risk	cause-specific cumulative hazard (13)
	cause-specific incidence (14)
Regression	mean (19)
Classification	class proportions (25)
Multivariate	mean per response (19)
	class proportions per response (25)
Unsupervised	none

In the table [Prediction Error], the error rate calculation for the five models is summarized. For Survival, it is based on Harrell's concordance-index using the cumulative hazard estimate as the values for comparison. For Competing Risk, Harrell's concordance-index uses the cause-specific cumulative-hazard estimate. For Regression, performance is based on mean-squared error. For classification, performance is based on the conditional and over-all misclassification rate. For the Multivariate and Unsupervised case, there is no prediction error implemented. See the [Theory and Specifications] section for more details on the mathematics of the prediction error for each model.

Family	Prediction Error

Survival	Harrell's C-index using cum-haz (5) and (6)
Competing Risk	Harrell's C-index using cause-specific cum-haz (15) and (6)
Regression	mean-squared error (20)
Classification	conditional and over-all misclassification rate (26) and (27)
Multivariate	none
Unsupervised	none

We conclude this section by giving examples of a mixed multivariate model, and an unsupervised model. The data set `mtcars`, available in the base distribution of R is used. The usual outcome for the data set is `mpg` (miles per gallon). First, we modify the data set to force `carb` (number of carburetors) and `cyl` (number of cylinders) to be categorical values. We produce a mixed model where all three of these variable are considered as outcomes. The code is given below:

```
# Multivariate mixed outcome: Motor Trend Car Road Tests.  Miles per
# gallon is the usual response, but the data set is modified to
# introduce categorical and ordinal variables.
mtcars.mod <- mtcars
mtcars.mod$cyl <- factor(mtcars.mod$cyl)
mtcars.mod$carb <- factor(mtcars.mod$carb, ordered=TRUE)
mtcars.mix <- rfsrc(cbind(carb, mpg, cyl) ~., data = mtcars.mod)
```

We also show various ways to implement an unsupervised model in the code below. It is possible to choose one, or more pseudo-responses at each split via the formula specification.

```
# Motor Trend Car Road Tests.  Miles per gallon is the usual response,
# but it is ignored below and the data set is taken to be responseless.
mtcars.unspv <- rfsrc(Unsupervised() ~., data = mtcars)
# The above is the equivalent of:
mtcars.unspv <- rfsrc(Unsupervised(1) ~., data = mtcars)
# Alternatively and equivalently:
mtcars.unspv <- rfsrc(data = mtcars)
# Choose two pseudo responses:
mtcars.unspv <- rfsrc(Unsupervised(2) ~., data = mtcars)
```

4. 4. Variable Selection

The package has two primary approaches for variable selection and identifying interactions between variables. The function `max.subtree()` extracts maximal subtree information from a forest [Ishwaran et al., 2010], [Ishwaran et al., 2011]. The function `vimp()` extracts variable importance information from a forest [Ishwaran et al., 2007].

4.1. 4.1. Minimal Depth and Maximal Subtrees

Minimal depth is a dimensionless order statistic that measures the predictiveness of a variable in a tree. It can be used to select variables in high-dimensional problems. It assesses the predictiveness of a variable by a depth calculation relative to the root node of a tree. A maximal subtree for an x-variable x is the largest subtree whose root node splits on x (i.e. all the parent nodes of $x's$ maximal subtree have nodes that split on variables other than x). There can be more than one maximal subtree for a variable. A maximal subtree will not exist if there are no splits on the variable. The shortest distance from the root of the tree to the root of the closest maximal subtree of x is the minimal depth of x. A smaller value corresponds to a more predictive variable. The mean of the minimal depth distribution is used as the threshold value for deciding whether an x-variable's minimal depth value is small enough for the x-variable to be classified as strong. An important fact of minimal depth is that it does not depend on a comparison of error rates, as does variable importance (described later), but is only dependent on the topology of the forest. See [Ishwaran et al., 2010] and [Ishwaran et al., 2011] for more details. The maximal subtrees for x_1 and the tree shown in the figure labeled [Tree Decision Boundary] are highlighted in blue in the figure labeled [Maximal Subtrees for x_1]. There are exactly two of them. The minimal depth for x_1 is equal to one.

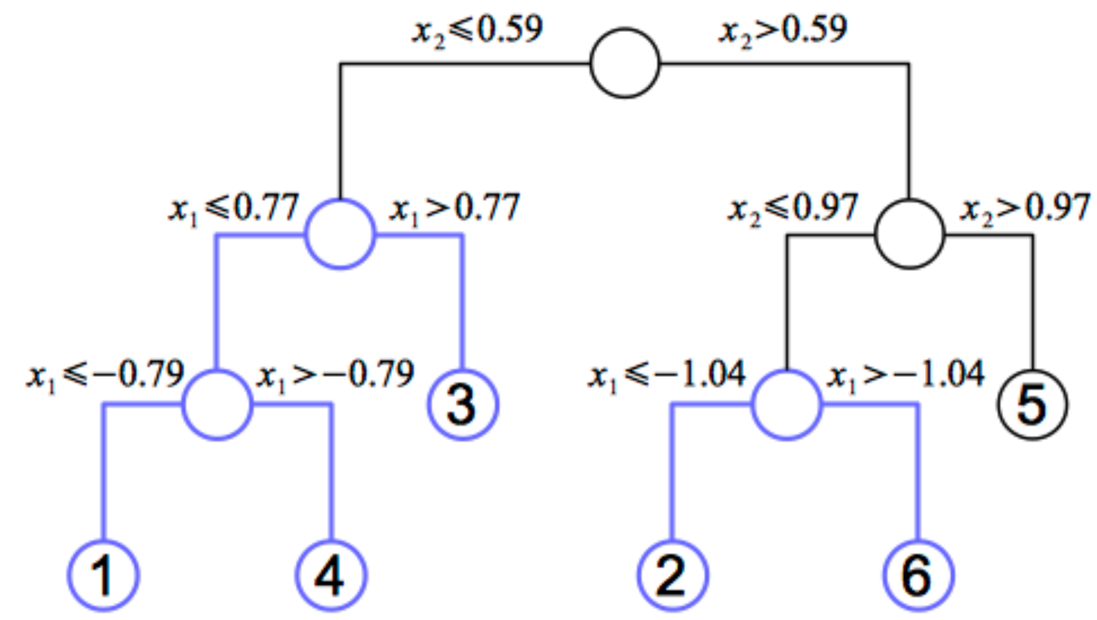

Maximal Subtrees for x_1

Examples of minimal depth and maximal subtrees follow:

```
# Survival Analysis with first and second order depths for all variables.
data(veteran, package = "randomForestSRC")
v.obj <- rfsrc(Surv(time, status) ~ . , data = veteran)
v.max <- max.subtree(v.obj)

# First and second order depths.
print(round(v.max$order, 3))

# The minimal depth is the first order depth.
print(round(v.max$order[, 1], 3))

# Strong variables have minimal depth less that or equal to the threshold.
print(v.max$threshold)

# This corresponds to the top set of variables above the threshold.
print(v.max$topvars)
```

4.2. 4.2. Variable Importance

The function `vimp()` extracts variable importance (VIMP) information [Ishwaran et al., 2007] from a forest for a single x-variable or group of x-variables. By default, VIMP is calculated for the training data, but the user can specify new test data for the VIMP calculation using the parameter `newdata`.

Action on the training data is as follows: The parameter `importance` allows four distinct ways to calculate VIMP. The default value of `importance="permute"` returns the Breiman-Cutler permutation VIMP as described in [Breiman, 2001]. For each tree, the prediction error on the OOB data is recorded. Then for a given x-variable x, OOB cases are randomly permuted in x and the prediction error is recorded. The VIMP for x for a tree is defined as the difference between the perturbed and unperturbed error rate for that tree. This is then averaged over all trees. If `importance="random"` is used, then OOB cases are assigned a daughter node randomly whenever a split on x is encountered in the tree. Again, the VIMP for x is defined as the difference between the perturbed and unperturbed error rate averaged over all trees. If the options `permute.ensemble` or `random.ensemble` are used, then VIMP is calculated by comparing the error rate for the perturbed OOB forest ensemble to the unperturbed OOB forest ensemble. Unlike the Breiman-Cutler measure, these last two VIMP ensembles do not measure the tree average effect of perturbing x, but rather the overall forest effect. See [Ishwaran et al., 2008] for further details.

Joint VIMP is requested using `joint=TRUE`. The joint VIMP is the importance for a group of variables when the group is perturbed simultaneously.

VIMP is completely dependent on prediction error. Although tempting, it is incorrect to assume VIMP measures the change in prediction error for a forest grown with and without a variable. For example, if two variables are highly correlated and both predictive, each can have large VIMP values. Removing one variable and regrowing the forest may affect the VIMP for the other variable (its value might get larger), but prediction error will likely remain unchanged. VIMP measures the change in prediction error on a fresh test case if x were not available, given that the original forest was grown using x. In practice, this often equals change in prediction error for a forest grown with and without x, but conceptually the two quantities are different. Examples follow:

```
# Classification example showcasing different vimp measures.
iris.obj <- rfsrc(Species ~ ., data = iris)

# Breiman-Cutler permutation vimp.
print(vimp(iris.obj)$importance)

# Breiman-Cutler random daughter vimp.
print(vimp(iris.obj, importance = "random")$importance)

# Breiman-Cutler joint permutation vimp.
print(vimp(iris.obj, joint = TRUE)$importance)
```

```
# Breiman-Cuter paired vimp.
print(vimp(iris.obj, c("Petal.Length", "Petal.Width"), joint=TRUE)$importance)
print(vimp(iris.obj, c("Sepal.Length", "Petal.Width"), joint=TRUE)$importance)
```

5. 5. Imputation

To handle data sets with missing data, the package provides two interfaces to impute missing values. If a predictive model is desired, then the user can create a forest with a call to `rfsrc()`. If all that is desired is a data set containing imputed values, without the need for a model, then a call to `impute()` will suffice. If no formula is specified, unsupervised splitting is implemented. The number of impute iterations is specified via the parameter `nimpute`. The number of impute iterations corresponds to the number of forests grown. When a predictive model is desired, only the final forest is retained. Note that two impute iterations were found to be the minimum necessary to produce reasonable imputed values.

On the first impute iteration, imputation is conducted in each internal node as the tree is grown. This in-node imputation is used to determine left and right daughter membership during the grow process. Note that imputed values are never used in split statistics on the first impute iteration. On the second, and subsequent impute iterations, in-node imputation is absent. Instead, the forest is grow as if there was no missing data, with missingness holes in the data plugged by the imputed values determined by the previous impute iteration. Imputation on the second and subsequent iteration only occurs in the terminal nodes, after the forest has been grown. The default setting for `rfsrc()` is `nimpute = 1`. The default setting for `impute()` is `nimpute = 2`.

Once a model has been created using `rfsrc()`, test data with or without missingness can be used with `predict.rfsrc()`. Values in the test data set are imputed based on the non-missing values in the training data set. Internally, both `rfsrc()` and `impute.rfsrc()` use variations of the missing data algorithm in [Ishwaran et al., 2008]. Setting `na.action = "na.impute"` imputes missing data (both x-variables and y-outcomes), while `na.action = "na.omit"` eliminates records with missingness and then grows the forest. Examples follow:

```
# Examples of survival imputation.

# Create a model via imputation.
data(pbc, package = "randomForestSRC")
pbc.d <- rfsrc(Surv(days, status) ~ ., data = pbc)

# Impute, using unsupervised splitting.
pbc2.d <- impute.rfsrc(data = pbc, nodesize = 1, nsplit = 10)
```

6. 6. Prediction

Predicted values are obtained by dropping test data down the forest after the model has been created. The overall error rate is returned if the test data contains a y-outcome. If no test data is provided, then the original training data is used and the code reverts to restoring the forest, associated terminal node statistics and ensembles. This is useful in that it gives the user the ability to extract outputs from the forest that were not asked for during model creation. The figure [Forest Decision Boundary] is an example of how prediction can be used to view the forest decision boundary imposed on the x-variables. Examples of traning, testing and restoration follow:

Training/testing scenario:

```
data(veteran, package = "randomForestSRC")
# Sample the data and create a training subset.
train <- sample(1:nrow(veteran), round(nrow(veteran) * 0.80))
# Train the model.
veteran.grow <- rfsrc(Surv(time, status) ~ ., veteran[train, ], ntree = 100)
# Test the model.
veteran.pred <- predict(veteran.grow, veteran[-train , ])
# Compare the results.
print(veteran.grow)
print(veteran.pred)
```

Restoration scenario:

```
# Train the model.
airq.obj <- rfsrc(Ozone ~ ., data = airquality)
# Restore the model and note identical results.
predict(airq.obj)
# Note that the two results are identical.
print(airq.obj)
# Retrieve an output that was not asked for in the original call.
prox <- predict(airq.obj, proximity = TRUE)$proximity
```

6.1. 6.1. outcome = "test"

If `outcome = "test"`, the training data is not used to calculate the terminal nodes statistics. Instead, the TNS are recalculated using the y-outcomes from the test set. This yields a modified predictor in which the invariant topology of the forest is based on the training data, but where the ensembles and predicted values are based on the test data. The terminal node statistics, ensembles and error rates are all calculated by bootstrapping the test data and using OOB individuals to ensure unbiased estimates.

Consider the the forest depicted previously in [Forest Decision Boundary] and the associated forest ensemble for the single test point at the origin. This original training data and ensemble are shown in the top left and top right of [outcome = "test"]. New test data, formed by reducing the radii of the blue and yellow classes is shown in the bottom left of the figure. The origin contains only two data points, namely one red and one green point. Using the new test data to populate terminal nodes, results in an ensemble that is missing the blue and yellow classes. This ensemble is shown in the bottom right of the figure.

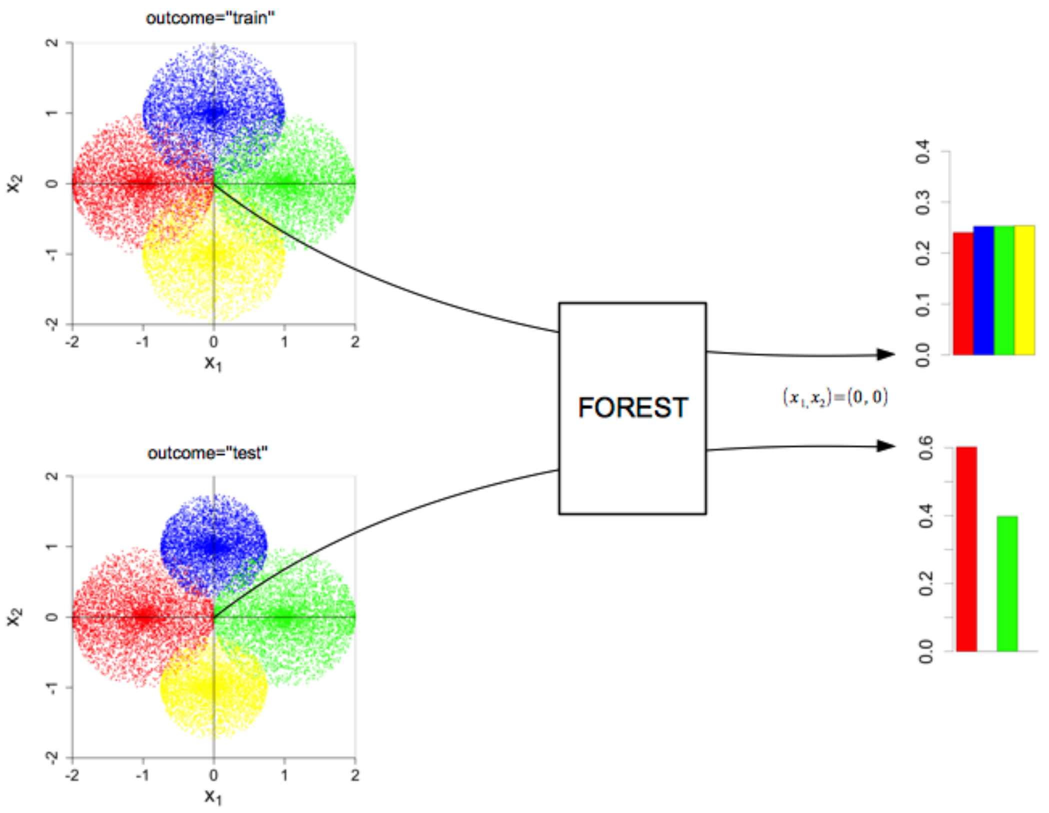

outcome = "test"

The function for creating the spheres is given in the section [Supplementary Code], and the higher level code used to produce the [outcome = "test"] figure follows:

```
## Four circular regions with equal class counts, touching at the origin.
## We suppress the X3 dimension in order to get a circle from a sphere.
circles <- get.spheres()[1, 2, 4]

## Grow the forest.
grow.result <- rfsrc(outcome ~ ., data = circles, importance = "none")

## Get a new test data set by restricting yellow and blue at the origin.
test.circles <- get.spheres(class.radius=c(1.0, 0.75, 1.0, 0.75))[1, 2, 4]

## Predict using the training data for terminal node statistics.
outcome.train <- predict(grow.result, newdata=test.circles)

## The origin (first row) will have roughly equal classes.
print(outcome.train$predicted[1, ])

## Predict using the test data for terminal node statistics.
outcome.train <- predict(grow.result, newdata=test.circles, outcome="test")

## The origin (first row) will only have red and yellow classes.
print(outcome.train$predicted[1, ])
```

6.2. 6.2. Pruning

There are a number of ways to limit the growth of trees in a model. The most obvious are the parameters `nodedepth` and `nodesize` discussed in the section [Node Depth and Node Size. Decreasing `nodedepth` or increasing `nodesize` will result in shallower trees. After a forest has been created, the user is able to prune trees back using the parameter `ptn.count`. This parameter represents the pseudo-terminal node count. It allows us to specify the desired number of pseudo-terminal nodes after a tree has been grown. This is not useful for Random Forests analysis per se, but it can be useful in other applications. Trees are flexible and adaptive nonparametric estimators and, as such, they represent ideal weak learners for implementing gradient boosting [Friedman, 2001]. Boosted trees for regression and classification, where exactly J-terminal nodes (for any integer value of J) are needed, is easily accomplished using the parameter `ptn.count`. This particular application of the `randomForestSRC` package is incorporated into the `boostmtree` package on CRAN [Ishwaran and Kogalur, 2016]. Pruning is accomplished by deleting terminal nodes from the maximum depth back toward the root until the desired pseudo-terminal node count is achieved. Daughter nodes are deleted in pairs. This results in a parent node becoming a pseudo-terminal node, after the daughter terminal nodes have been deleted at the current maximum depth.

7. 7. Hybrid Parallel Processing

This package has the capabilities of non-trivial parallel processing with massive scalability. Growing a forest has a recursive component

as was shown in the [Recursive Algorithm]. But it is also an iterative process that is repeated `ntree` times. This fact is depicted in the figure [Iterative Process]. The entry point is a user-defined R script that initiates the grow call `rfsrc()`. This function pre-processes the data set, and calls a grow function in the package's C library, which grows the forest. The C function grows a single tree, adds the resulting tree-specific ensemble(s) to the forest ensemble(s), and continues this process for `ntree` iterations. The C function then returns the forest ensemble(s) to the R function, which post-processes the results. It is advantageous to parallelize this iterative process. Such situations are often called "pleasingly parallel". The default pre-compiled binaries, available on CRAN, should execute in parallel. If not, the package has been written to be easily compiled to accommodate parallel execution. As a prerequisite, the host architecture, operating system, and installed compilers must support it. OpenMP and Open MPI compilers must be available on the host system. If this is the case, it is possible to compile the source code to enable parallel processing.

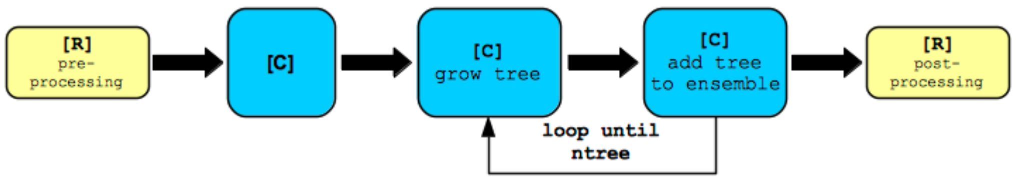

Forest Growth as an Iterative Process

A graphic showing one mode of parallel execution (OpenMP) on a typical desktop/laptop computer is shown in the figure [Shared Memory Parallel Processing]. In this example, the hardware on the left of the figure is a Macbook Pro (2015 model) with two quad-core CPUs, and 16GB of RAM. This hardware allows eight threads to execute simultaneously. The memory is shared among all cores, and this configuration is typical of symmetric multiprocessing (SMP) where two or more identical processors connect to a shared memory bank. Most reasonably configured multi-core notebooks and desktops are ideal hosts for `randomForestSRC` and OpenMP parallel execution. The right side of [Shared Memory Parallel Processing] shows the software implementation of the OpenMP model. The R code reads the data set, pre-processes the data set, calls the C code and requests a forest of `ntree` trees. When OpenMP parallel execution is in force, the C code grows trees simultaneously, rather than iteratively. Each core is tasked with growing a tree. Once a core completes growing a tree, the forest ensemble values are incremented with the contribution of that tree, and the core is re-tasked with growing another tree. This continues until the entire forest is grown. Control is then returned to the R code which does post-processing of the results. On the hardware shown in the figure, it is possible to grow eight trees at a time. Under ideal conditions, the elapsed time to grow a forest should decrease linearly and be close to a factor of eight.

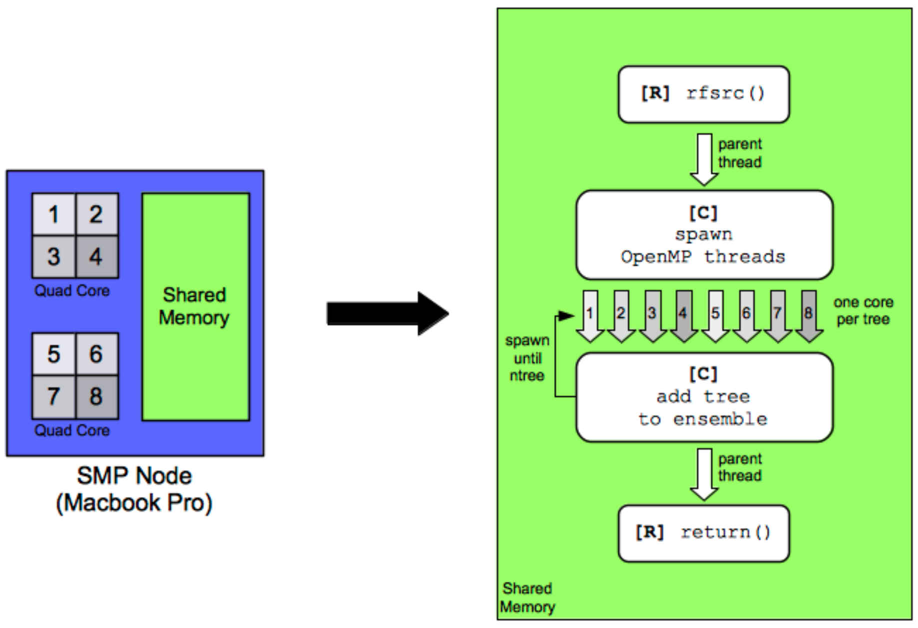

OpenMP: Shared Memory Parallel Processing

In order to give the user some flexibility, core usage can be controlled via R options. First, the user needs to determine the number of cores on the machine. This can be done within an R session by loading the `parallel` package and issuing the command `detectCores()`. It is possible to set the numbers of cores accessed during OpenMP parallel execution at the start of every R session by issuing the command `options(rf.cores = x)`, where `x` is the number of cores. If `x` is a negative number, the package will access the maximum number of cores on the machine. The `options` command can also be placed in the users `.Rprofile` file for convenience. Alternatively, one can initialize the environment variable `RF_CORES` in the user's command shell environment.

Gains in performance on a desktop (or a single SMP node) are limited by the number of cores available on the hardware. The OpenMP parallel model shown in [Shared Memory Parallel Processing] that relies on an underlying iterative process was chosen for its suitability in accommodating shared memory systems and its ease of implementation. Model creation is computationally intense and the OpenMP parallel processing model allows us to take full advantage of the hardware available.

Message Passing Interface (MPI), an alternative model to OpenMP is suitable for distributed memory systems, in which the number of cores available can be much greater than that of a desktop computer. It is possible to run `randomForestSRC` on appropriately designed and configured clusters, allowing for massive scalability. An ideal cluster for the package is made up of tightly connected SMP nodes. Each SMP node has its own memory, and does not share this memory with other nodes. Nodes, however, do communicate with each other using MPI.

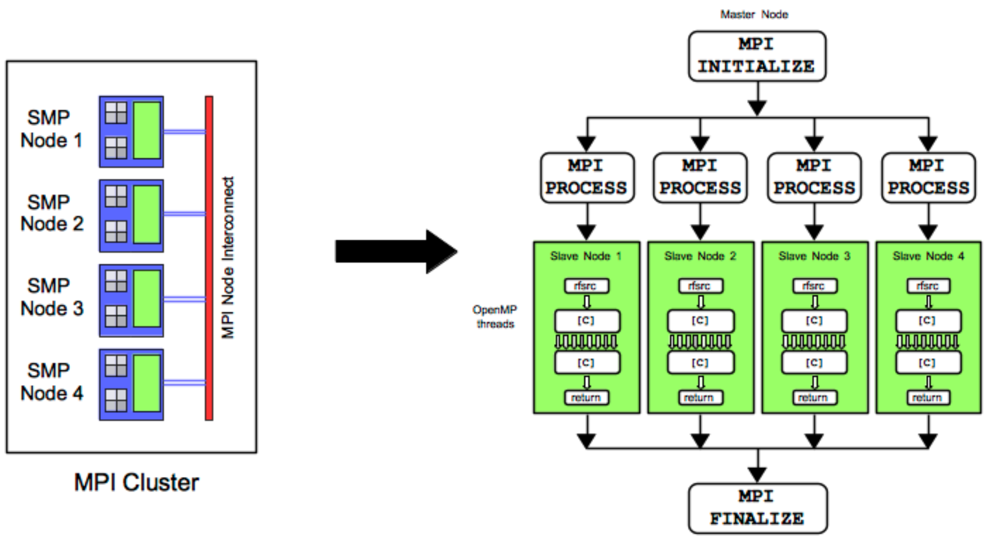

Hybrid: MPI/OpenMP Parallel Processing

In the figure [MPI/OpenMP Parallel Processing], a generic cluster with four SMP nodes is depicted. Each node can be considered to be similar to a desktop computer. However, in a cluster setting, each node is tightly supervised and controlled by a top-level layer of cluster software that handles job scheduling, and resource management. On the left of the figure, four nodes are connected via a network that facilitates MPI communications across nodes. The right of the figure depicts the software implementation of hybrid parallel processing using the `randomForestSRC` package. A primary/replica framework is chosen whereby a primary node on the cluster is tasked with growing the forest. The primary node divides the forest into sub-forests. If the size of the forest is `ntree`, then each replica in this example is tasked with growing a sub-forest of size `ntree/4`. Hybrid parallel processing is dependent on the `Rmpi` package. This package must be available. If it is not, it must compiled and installed from the source code available on CRAN. The `Rmpi` is a mature package and provides flexible low level functionality. Other explicitly parallel general-use packages on CRAN such as `snow`, `snowfall`, and `foreach` provide higher level user friendly functions that, while potentially useful, were considered not as flexible and customizable in a cluster environment.

In the hybrid example that follows, `Rmpi` is loaded on the primary node, and MPI processes are then spawned on each replica. Disregarding the fact that the replicas can communicate with the primary using MPI, each replica acts like an SMP node in [Shared Memory Parallel Processing]. A replica initiates concurrent OpenMP threads across it cores, and grows its sub-forest using shared memory OpenMP parallel processing. It is possible to grow many more trees concurrently on a cluster, because the forest is spread among many more cores than are available on a single node, or desktop computer. In [MPI/OpenMP Parallel Processing], the forest uses 32 cores concurrently. In an ideal environment, the elapsed time to grow the original forest will be reduced by a factor of 32. After all replicas have completed growing their sub-forests, they return their data to the primary process, via MPI messages, and the replicas terminate. On a single SMP node (equivalent to a desktop computer), a thread on a core starts, grows a tree, terminates, and repeats this process until all trees have been grown. A core is either waiting for a task, working on a task, or has completed a task. A task, in this case, is the growing of a tree. On a cluster, a replica node starts, grows a sub-forest, and terminates. It repeats this process until all sub-forests requested by the primary node have been grown. A replica is either waiting for a sub-forest, working on a sub-forest, or has completed a sub-forest.

As proof of concept, and for benchmarking purposes we ran the package on two clusters available to us. The first cluster was the Learner Research Institute High Performance Cluster (LRI-HPC) at the Cleveland Clinic. This system runs Simple Linux Utility for Resource Management (SLURM) supervisor software. It is a Linux-based cluster consisting of twenty nodes with ten cores per node. Each node has 64GB of shared memory. The second cluster was the [Pegasus 2] installation at the High Performance Computing Group at the Center for Computational Science (HPC CCS) at the University of Miami. This system has 10,000 cores spread across over 600 nodes. The system also runs a flavour of Linux but uses Platform LSF as the job scheduler. The different job schedulers for the two clusters required minor customization of the calling shell scripts. For our benchmarks, we analyzed a simulated survival data set with `n=3000`, `p=30`, and `ntree=1024`, with the default value of `importance="permute"`. We ran the package in serial mode and scaled up to 1024 cores across 64 nodes. The results are shown in this figure. In serial mode, growing 1024 trees iteratively (using one core) took about 330 minutes. The red points all reflect OpenMP parallel processing, on one node. These results would be similar to that of a desktop computer. Pegasus 2 has 16 cores per node. When using all cores on one node were used, elapsed time decreased to about 25 minutes. This reflects a decrease in a factor of 13 from the serial time. The theoretical maximum decrease would be closer to a factor of 16. The results would be similar to that of running the analysis on desktop computer with 16 cores. Next, hybrid parallel processing using the primary/replica framework was implemented. Two replica nodes were tasked, then three, and so on up to 64 nodes. At 64 nodes, total core usage was 1024. At this number, the cluster was growing all 1024 trees simultaneously. The data points reflecting hybrid parallel processing are in blue. Linear decreases in elapsed time were observed. This indicates that the package is capable of massive scalability. The primary and replica code harnesses are given in the section [Supplementary Code].

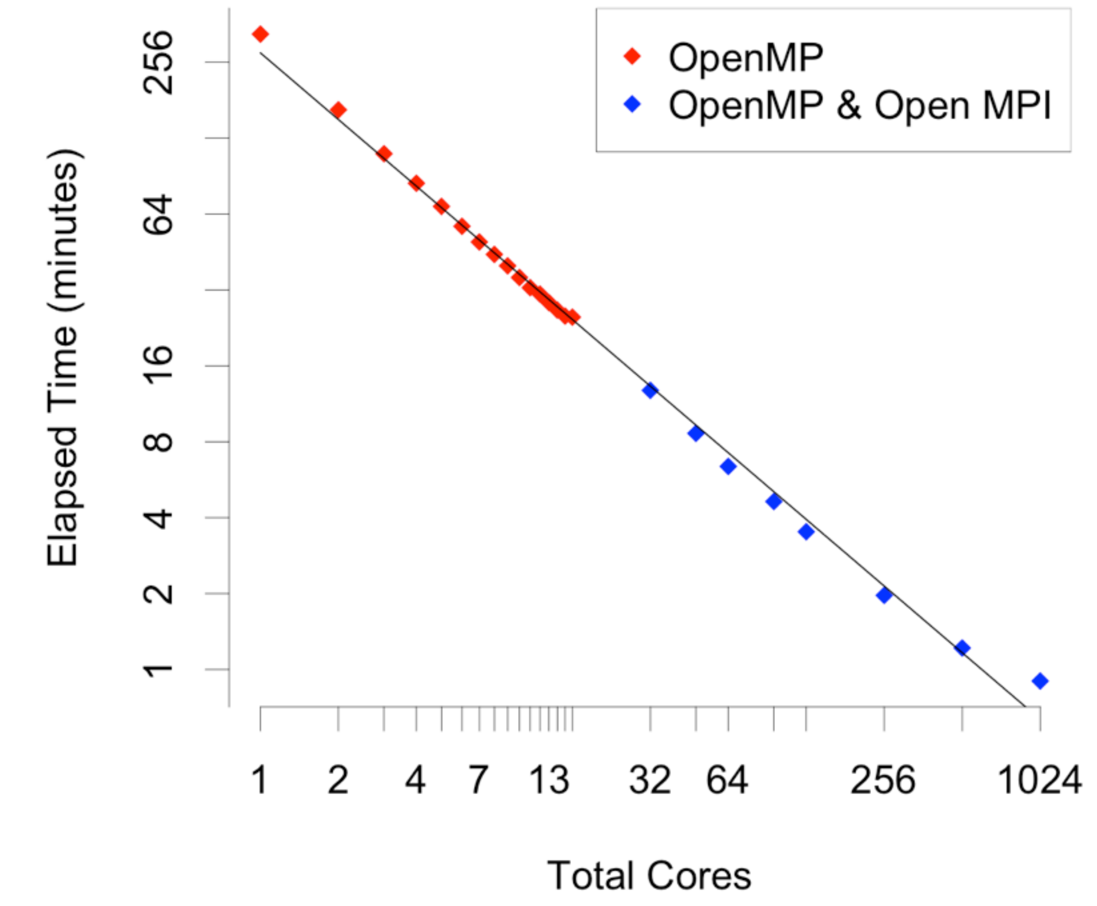

Benchmark of Hybrid Parallel Processing

8. 8. Theory and Specifications

8.1. 8.1. Survival

There are two split rules that can be used to grow survival trees. In this model, the response or y-outcome associated with individual i is a pair of values specifying a non-negative survival time and censoring information. Denote the response for i by $Y_i = (T_i, \delta_i)$. The individual is said to be right censored at time T_i if $\delta_i = 0$ and is said to have died at time T_i if $\delta_i = 1$. In the case of death, T_i will be referred to as an event time, and the death as an event. An individual i who is right censored at T_i simply means that the individual is known to have been alive at T_i, but the exact time of death is unknown.

Log-rank splitting

The log-rank test for splitting survival trees is a well established concept [Segal, 1988]. It has been shown to be robust in both proportional and non-proportional hazard settings [Leblanc and Crowley, 1993] Note that a proposed split of h on a real value x is of the form $x \le c$ and $x > c$. Such a split defines left and right daughter membership. The split that maximizes survival differences between the two daughter nodes is the best split. Let $t_1 < t_2 < \cdots < t_m$ be the distinct times of death in the parent node h, and let $d_{k,l}$ and $Y_{k,l}$ equal the number of deaths and individuals at risk respectively at time t_k in the left daughter node for $k \in \{1, \ldots, m\}$. Similarly let $d_{k,r}$ and $Y_{k,r}$ refer to the right daughter node. Note that $Y_{k,s}$ is the number of individuals in daughter $s \in \{l, r\}$ who are alive at time t_k, or who have an event (death) at time t_k. More precisely,

$$Y_{k,l} = \#\{i : T_i \ge t_k,\ x_i \le c\}, \qquad Y_{k,r} = \#\{i : T_i \ge t_k,\ x_i > c\},$$

where x_i is the value of x for individual $i = 1, \ldots, n$. Define $Y_k = Y_{k,l} + Y_{k,r}$ and $d_k = d_{k,l} + d_{k,r}$. Let n_s be the total number of observations in daughter s, Thus, $n = n_l + n_r$, where $n_l = \#\{i : x_i \le c\}$ and $n_r = \#\{i : x_i > c\}$. The log-rank test for a split at the value c for an x-variable x is

$$L(x, c) = \frac{\displaystyle\sum_{k=1}^{m} \left(d_{k,l} - Y_{k,l} \frac{d_k}{Y_k} \right)}{\sqrt{\displaystyle\sum_{k=1}^{m} \frac{Y_{k,l}}{Y_k} \left(1 - \frac{Y_{k,l}}{Y_k} \right) \left(\frac{Y_k - d_k}{Y_k - 1} \right) d_k}}. \tag{1}$$

The value $|L(x, c)|$ is a measure of node separation. Larger values of $|L(x, c)|$ imply a greater the difference between the two groups, and the better the split. In particular, the best split at node h is determined by finding the x-variable x^* and split value c^* such that $|L(x^*, c^*)| \ge |L(x, c)|$ for all x and c.

Log-rank score splitting

The log-rank score test for splitting survival trees is described in [Hothorn and Lausen (2003)]. In this rule, assume the x-variable x has been ordered so that $x_1 \le x_2 \le \cdots \le x_n$. Now, compute the ``ranks'' for each survival time T_j where $j \in \{1, \ldots, n\}$. Thus,

$$a_j = \delta_j - \sum_{k=1}^{\Gamma_j} \frac{\delta_k}{n - \Gamma_k + 1},$$

where $\Gamma_k = \#\{t : T_t \leq T_k\}$. Note that Γ_k is the index of the order for T_k. The log-rank score test is defined as

$$S(x, c) = \frac{\sum_{x_k \leq c} (a_j - n_l \bar{a})}{\sqrt{n_l \left(1 - \frac{n_l}{n}\right) s_a^2}}, \tag{2}$$

where $\bar{a}$ and s_a^2 are the sample mean and sample variance of $\{a_j : j = 1, \ldots, n\}$. Log-rank score splitting defines the measure of node separation by $|S(x, c)|$. Maximizing this value over x and c yields the best split.

Terminal node estimators

The survival estimate associated with a terminal node is provided by the Kaplan-Meier (KM) estimator [Kaplan and Meier (1958)]. Again let $t_1 < t_2 < \cdots < t_m$ be the distinct death times in the terminal node h, and let d_k and Y_k equal the number of deaths and individuals at risk respectively at time t_k in h. Then the Kaplan-Meier estimator is

$$\hat{S(t)} = \prod_{t_k \leq t} \left(1 - \frac{d_k}{Y_k}\right). \tag{3}$$

The cumulative hazard estimate (CHE) associated with a terminal node is provided by the Nelson-Aalen estimator and is calculated as

$$\hat{H(t)} = \sum_{t_k \leq t} \frac{d_k}{Y_k}. \tag{4}$$

If there are H terminal nodes in a tree, there are H such estimates. Let individual i have feature $\mathbf{X}_i$ and reside in terminal node h. The CHE terminal node statistic will be denoted by

$$\hat{H(t|\mathbf{X}_i)} = \hat{H_h}(t), \text{ if } \mathbf{X}_i \in h.$$

To produce the OOB ensemble for individual i, the CHE terminal node statistic is averaged over all $\texttt{ntree}$ trees. Let $\hat{H_b}(t|X)$ denote the CHE estimate for tree $b \in \{1, \ldots, \texttt{ntree}\}$. Define $I_{i,b} = 1$ if individual i is an OOB individual for b, otherwise set $I_{i,b} = 0$. The OOB ensemble CHE for individual i is

$$\hat{H_e^*}(t|\mathbf{X}_i) = \frac{\sum_{b=1}^{\texttt{ntree}} I_{i,b} \hat{H_b}(t|\mathbf{X}_i)}{\sum_{b=1}^{\texttt{ntree}} I_{i,b}}.$$

Note that the OOB ensemble is obtained by averaging over only those bootstraps in which individual i is is OOB.

An example of a survival forest follows:

```
# Veteran's Administration Lung Cancer Trial. Randomized
# trial of two treatment regimens for lung cancer.
data(veteran, package = "randomForestSRC")
v.obj <- rfsrc(Surv(time, status) ~ ., data = veteran, ntree = 100)
## Plot the error.
plot(v.obj)
## Plot the survival estimates.
plot.survival(v.obj)
```

Prediction error

Prediction error for survival models is measured by $1 - C$, where C is Harrell's [] concordance index. Prediction error is between 0 and 1, and measures how well the predictor correctly ranks two random individuals in terms of survival. Unlike other measures of survival performance, Harrell's C-index does not depend on choosing a fixed time for evaluation of the model and specifically takes into account censoring of individuals [May et al., (2004)] The method has quickly become quite popular in the literature as a means for assessing prediction performance in survival analysis settings. See [Kattan et al., 1988] and references therein.

To compute the concordance index, it is necessary to define what constitutes a worse predicted outcome. Let $t_1^*, \ldots, t_M^*$ denote all unique times in the data. Define the predicted outcome for individual i to be

$$\mathcal{M}_i = \sum_{k=1}^{M} \hat{H_e^*}(t_k^*|\mathbf{X}_i). \tag{5}$$

Individual i is said to have a worse predicted outcome than individual j if $\mathcal{M}_i > \mathcal{M}_j$ The concordance error rate is computed as

follows:

1. Form all possible pairs of observations over all the data.
2. Omit those pairs where the shorter event time is censored. Also, omit pairs (i, j) if $T_i = T_j$ unless $(\delta_i = 1, \delta_j = 0)$ or $(\delta_i = 0, \delta_j = 1)$ or $(\delta_i = 1, \delta_j = 1)$. The last restriction only allows ties in event times if at least one of the observations is a death. Let the resulting pairs be denoted by $\mathbb{S}$. Let $permissible = |\mathbb{S}|$.
3. If $T_i \neq T_j$, count 1 for each $s \in \mathbb{S}$ in which the shorter time had the worse predicted outcome.
4. If $T_i \neq T_j$, count 0.5 for each $s \in \mathbb{S}$ in which $\mathcal{M}_i = \mathcal{M}_j$.
5. If $T_i = T_j$, count 1 for each $s \in \mathbb{S}$ in which $\mathcal{M}_i = \mathcal{M}_j$.
6. If $T_i = T_j$, count 0.5 for each $s \in \mathbb{S}$ in which $\mathcal{M}_i \neq \mathcal{M}_j$.
7. Let $concordance$ denote the resulting count over all permissible pairs. Define the concordance index C as

$$C = \frac{concordance}{permissible}. \tag{6}$$

8. The error rate is $Err = 1 - C$. Note that $0 \leq Err \leq 1$ and that $Err = 0.5$ corresponds to a procedure doing no better than random guessing, whereas $Err = 0$ indicates perfect prediction.

8.2. 8.2. Competing Risk

Competing risks occur in survival analyses when the occurrence of one event precludes the occurrence of the remaining set of possible events. Individuals subject to competing risks are observed from study entry to the occurrence of the event of interest. This is a competing event though often, before the individual can experience one of the events, that person is right censored.

Formally, let T_i^o be the event time for the ith subject, $i = 1, \ldots, N$, and let δ_i^o be the event type, $\delta_i^o \in \{1, \ldots, J\}$, where $J \geq 1$. Let C_i^o denote the censoring time for individual i such that the actual time of event T_i^o is unobserved and one only observes $T_i = \min(T_i^o, C_i^o)$ and the event indicator $\delta_i = \delta_i^o I(T_i^o \leq C_i^o)$. When $\delta_i = 0$, the individual is said to be censored at T_i, otherwise if $\delta_i = j > 0$, the individual is said to have an event of type j at time T_i. Denote the observed response for i as $Y_i = (T_i, \delta_i)$. The cause-specific hazard function (cs-H) for event j given a P-dimensional feature $\mathbf{X}_i$ is

$$\alpha_j(t|\mathbf{X}_i) = \lim_{\Delta t \to 0} \frac{\mathbb{P}\{t \leq T^o \leq t + \Delta t, \delta^o = j | T^o \geq t, \mathbf{X}_i\}}{\Delta t} = \frac{f_j(t|\mathbf{X}_i)}{S(t|\mathbf{X}_i)}. \tag{7}$$

Here $S(t|\mathbf{X}_i) = \mathbb{P}\{T^o \geq t|\mathbf{X}_i\}$ is the event-free survival probability function given $\mathbf{X}_i$. The cs-H describes the instantaneous risk of event j for subjects that currently are event-free. The probability of an event is determined using the cause-specific cumulative incidence function (cs-CIF), defined as the probability of experiencing an event of type j by time t; i.e. $F_j(t|\mathbf{X}_i) = \mathbb{P}\{T^o \leq t, \delta^o = j|\mathbf{X}_i\}$. The cs-CIF and cs-H are related according to

$$F_j(t|\mathbf{X}_i) = \int_0^t S(s - |\mathbf{X}_i)\alpha_j(s|\mathbf{X}_i)\,\mathrm{d}s = \int_0^t \exp\left(-\int_0^s \sum_{l=1}^J \alpha_l(u|\mathbf{X}_i)\mathrm{d}u\right)\alpha_j(s|\mathbf{X}_i)\,\mathrm{d}s. \tag{8}$$

See [Gray (1988)] for an analysis of the cs-CIF.

With these two equations, it is possible to describe the two split rules that can be used to grow competing risk trees. Again, for notational convenience these rules are described for the root node using the entire learning data, and for splits on real valued x-variables. However, the algorithm extends to any tree node, to bootstrap data, and to splits on categorical x-variables.

As before, let $T_i, \delta_i)_{1 \leq i \leq n}$ denote the pairs of survival times and event indicators. Let \(t_1c\) for a continuous x-variable x. Such a split forms two daughter nodes containing two new sets of competing risk data. The subscripts l and r refer to the left and right daughter node, respectively, and denote $\alpha_{jl}(t)$ and $\alpha_{jr}(t)$ for the cause-j specific hazard rate, given by (7), in the left and the right daughter node. Similarly define $F_{jl}(t)$ and $F_{jr}(t)$ to be the cs-CIF, given by (8), for the left and the right daughter node.

The number of individuals at risk at time t in the left and right daughter nodes are $Y_l(t)$ and $Y_r(t)$, where

$$Y_l(t) = \sum_{i=1}^n I(T_i \geq t, x_i \leq c), \qquad Y_r(t) = \sum_{i=1}^n I(T_i \geq t, x_i > c),$$

and x_i is the value the x-variable assumes for individual $i = 1, \ldots, n$. The number of type j events at time t for the left and right daughters is

$$d_{j,l}(t) = \sum_{i=1}^n I(T_i = t, \delta_i = j, x_i \leq c), \qquad d_{j,r}(t) = \sum_{i=1}^n I(T_i = t, \delta_i = j, x_i > c).$$

The total number of individuals who are risk at time t, and the total number of type j events at time t in the parent node are given by

$$Y(t) = Y_l(t) + Y_r(t), \tag{9}$$
$$d_j(t) = d_{j,l}(t) + d_{j,r}(t). \tag{10}$$

Define also t_m, t_{m_l}, t_{m_r} to be the largest time on study in the parent node and the two daughters, respectively.

Log-rank splitting

The first competing risk split rule is the log-rank test. This is a test of the null hypothesis $H_0 : \alpha_{jl}(t) = \alpha_{jr}(t)$ for all $t \le \tau$. The test is based on the weighted difference of the cause-specific Nelson-Aalen estimates in the two daughter nodes. Specifically, for a split at the value c for variable x, the split rule is

$$L_j^{\mathrm{LR}}(x, c) = \frac{1}{\sigma_j^{\mathrm{LR}}(x, c)} \sum_{k=1}^{m} W_j(t_k) \left(d_{j,l}(t_k) - \frac{d_j(t_k) Y_l(t_k)}{Y(t_k)} \right),$$

where the variance estimate is given by

$$(\sigma_j^{\mathrm{LR}}(x, c))^2 = \sum_{k=1}^{m} W_j(t_k)^2 d_j(t_k) \frac{Y_l(t_k)}{Y(t_k)} \left(1 - \frac{Y_l(t_k)}{Y(t_k)} \right) \left(\frac{Y(t_k) - d_j(t_k)}{Y(t_k) - 1} \right).$$

Time-dependent weights $W_j(t) > 0$ are used to make the test more sensitive to early or late differences between the cause-specific hazards. The choice $W_j(t) = 1$ corresponds to the standard log-rank test which has optimal power for detecting alternatives where the cause-specific hazards are proportional. In practice, $W_j(t) = W_j$. That is, the weights are constant and not time-dependent. Furthermore, the cause-specific split rules across the event types are combined to arrive at the composite log-rank competing split rule that is implemented in the package and given by

$$L^{\mathrm{LR}}(x, c) = \frac{\sum_{j=1}^{J} (\sigma_j^{\mathrm{LR}}(x, c))^2 L_j^{\mathrm{LR}}(x, c)}{\sqrt{\sum_{j=1}^{J} (\sigma_j^{\mathrm{LR}}(x, c))^2}}. \tag{11}$$

The best split is found by maximizing $|L^{\mathrm{LR}}(x, c)|$ over x and c.

Modified log-rank splitting

The cause-j specific log-rank split rule (11) is useful if the main purpose is to detect variables that affect the cause-j specific hazard. It may not be optimal if the purpose is also prediction of cumulative event probabilities. In this case better results may be obtained with split rules that select variables based on their direct effect on the cumulative incidence. For this reason the second split rule is modeled after Gray's test [Gray (1988)], which tests the null hypothesis $H_0 : F_{jl}(t) = F_{jr}(t)$ for all $t \le \tau$. See [Ishwaran et al. (2014)] for additional details in using the modified risk set defined by

$$Y_j^*(t) = \sum_{i=1}^{n} I\Big(T_i \ge t \cup \big(T_i < t \cap \delta_i \ne j \cap C_i^o > t \big) \Big).$$

This equals the number of individuals who have not had an event prior to t in addition to those individuals who have experienced an event $j^{'} \ne j$ prior to t, but who are not censored. The split rule based on the score statistic which uses the modified risk sets, is denoted $L_j^{\mathrm{G}}(x, c)$ and given by substituting Y_j^* for Y_j and Y_{jl}^* for Y_l in (11). Note that if the censoring time is not known for those cases that have an event before the end of follow-up, the largest observed time is used, and the statistic $L_j^{\mathrm{G}}(x, c)$ is still a good (and computationally efficient) approximation of Gray's test statistic, see [Section 3.2, Fine, Jason P and Gray, Robert J (1999)].

Thus the composite modified log-rank competing split rule is given by

$$L^{\mathrm{G}}(x, c) = \frac{\sum_{j=1}^{J} (\sigma_j^{\mathrm{G}}(x, c))^2 L_j^{\mathrm{G}}(x, c)}{\sqrt{\sum_{j=1}^{J} (\sigma_j^{\mathrm{G}}(x, c))^2}}. \tag{12}$$

The best split is found by maximizing $|L^{\mathrm{G}}(x, c)|$ over x and c.

Terminal node estimators

Let $t_1 < t_2 < \cdots < t_m$ be the distinct non-censored event times in the terminal node h, and let $d_j(t_k)$ equal the number of events of type j at time t_k. Let $Y(t_k)$ be the number of bootstrap cases at risk at time t_k. Both of these are is in (9) and (10). Let individual i have feature $\mathbf{X}_i$ and reside in terminal node h. Denote the number of events of type j in $[0, t_j]$ as

$$N_j(t|\mathbf{X}_i) = \sum_{t_k \le t} d_j(t|\mathbf{X}_i).$$

The cause-specific hazard for individual i in node h and event j is defined by $\alpha_j(t|\mathbf{X}_i)$ and given by (7). The cause-specific cumulative hazard function (cs-CHF) is given by

$$H_j(t|\mathbf{X}_i) = \int_0^t \alpha_j(s|\mathbf{X}_i) ds = \int_0^t \frac{dN_j(s|\mathbf{X}_i)}{Y(t_k|\mathbf{X}_i)}.$$

The Nelson-Aalen estimate for the cs-CHF is given by

$$\hat{H_j}(t|\mathbf{X}_i) = \sum_{t_k<t} \hat{\alpha_j}(t_k|\mathbf{X}_i) = \sum_{t_k<t} \frac{d_j(t_k|\mathbf{X}_i)}{Y(t_k|\mathbf{X}_i)}. \tag{13}$$

The cs-CIF is given by (8) and can be written as

$$F_j(t|\mathbf{X}_i) = \int_0^t S(s-|\mathbf{X}_i)\alpha_j(s|\mathbf{X}_i)\,\mathrm{d}s = \int_0^t S(s-|\mathbf{X}_i)\frac{dN_j(s|\mathbf{X}_i)}{Y(s|\mathbf{X}_i)}.$$

We have adopted the estimator for $F_j(t)$ given in [Equation 2.3, Gray (1988)]. Namely,

$$\hat{F_j}(t|\mathbf{X}_i) = \sum_{t_k<t} \hat{S}(t_{k-1}|\mathbf{X}_i)\frac{d_j(t_k|\mathbf{X}_i)}{Y(t_k|\mathbf{X}_i)}, \tag{14}$$

where $\hat{S}(t)$ is given by (3). The predicted value for event j is the integrated cs-CIF

$$\mathcal{M}_j = \int_0^\tau F_j(s)ds.$$

Here τ is a fixed time point. We use $\tau = t_M$, the maximum observed event time. The value $\mathcal{M}_j$ can be interpreted as the *expected number of life years lost* and represents a measure of mortality. This is also called the cause-j mortality, is denoted by $\mathcal{M}_j$ and given by

$$\hat{\mathcal{M}_j} = \int_0^\tau \hat{F_j}(s)ds = \sum_{t_{k+1}\le\tau} \hat{F_j}(t_k)[t_{k+1}-t_k]. \tag{15}$$

Prediction error

The cause-specific error rate for each event j is estimated using the concordance index protocol resulting in equation (6). Let $\hat{\mathcal{M}}_{i,j}$ denote the cause-j mortality for case i. We say that case i has a higher risk of event j than case i' if $\hat{\mathcal{M}}_{i,j} > \hat{\mathcal{M}}_{i',j}$. Applying equation (6) results in a concordance index C_j and an error rate $Err_j = 1 - C_j$. An example of a competing risk call follows:

```
# Women's Interagency HIV Study (WIHS).  Competing risk
# data set involving AIDS in women.
data(wihs, package = "randomForestSRC")
wihs.obj <- rfsrc(Surv(time, status) ~ ., data=wihs, nsplit=3, ntree=100)
#  Plot the ensemble cs-CIF, and cs-CHF.
plot.competing.risk(wihs.obj)
```

8.3. 8.3. Regression

There are three split rules that can be used to grow regression trees. In this model, the y-outcome associated with an individual i is a single real value. Denote the response for i by $Y_i \in \mathbb{R}$.

Weighted variance splitting

Suppose the proposed split for the root node is of the form $x \le c$ and $x > c$ for a continuous x-variable x, and a split value of c. The split rule is

$$\theta(x,c) = \frac{n_l}{n} \times \frac{1}{n_l}\sum_{x_i\le c}(Y_i - \bar{Y}_l)^2 + \frac{n_r}{n} \times \frac{1}{n_r}\sum_{x_i>c}(Y_i - \bar{Y}_r)^2, \tag{16}$$

where the subscripts l and r indicate left and right daughter node membership respectively. Then

$$\bar{Y}_l = \frac{1}{n_l}\sum_{x_i\le c} Y_i, \quad \bar{Y}_r = \frac{1}{n_r}\sum_{x_i>c} Y_i$$

are the means within each of the daughters, and n_l and n_r are the number of cases in the left and right daughter respectively such that $(n = n_l + n_r)$. The goal is to find x and c to minimize $\theta(x,c)$.

Unweighted variance splitting

In this rule, it is necessary to minimize

$$\theta(x, c) = \frac{1}{n_l} \sum_{x_i \le c} (Y_i - \bar{Y}_l)^2 + \frac{1}{n_r} \sum_{x_i > c} (Y_i - \bar{Y}_r)^2. \tag{17}$$

Heavy weighted variance splitting

In this rule, it is necessary to minimize

$$\theta(x, c) = \left(\frac{n_l}{n}\right)^2 \times \frac{1}{n_l} \sum_{x_i \le c} (Y_i - \bar{Y}_l)^2 + \left(\frac{n_r}{n}\right)^2 \times \frac{1}{n_r} \sum_{x_i > c} (Y_i - \bar{Y}_r)^2. \tag{18}$$

Terminal node estimators

The predicted value for a terminal node is the mean value of the cases within that node. Let individual i have feature $\mathbf{X}_i$. and reside in terminal node h. The terminal node predicted value for a node h is given by

$$\hat{f_h} = \frac{1}{n_h} \sum_{\mathbf{X}_i \in h} Y_i. \tag{19}$$

To produce the OOB predicted value for individual i this quantity is averaged over all $\mathtt{ntree}$ trees. Let $\hat{f_b}(\mathbf{X}_i)$ denote the predicted value for tree $b \in \{1, \ldots, \mathtt{tree}\}$. As before, $I_{i,b} = 1$ if individual i is an OOB individual for $b \in \{1, \ldots, \mathtt{ntree}\}$. Otherwise set $I_{i,b} = 0$. The OOB predicted value for individual i is

$$f_e^*(\mathbf{X}_i) = \frac{\sum_{b=1}^{\mathtt{ntree}} I_{i,b} \hat{f_b}(\mathbf{X}_i)}{\sum_{b=1}^{\mathtt{ntree}} I_{i,b}}.$$

Prediction error

The estimated prediction error is given by

$$Err = \frac{1}{n} \sum_{i=1}^{N} (Y_i - f_e^*(\mathbf{X}_i))^2. \tag{20}$$

An example of a regression forest follows:

```
# New York air quality measurements. Mean ozone in parts per billion.
airq.obj <- rfsrc(Ozone ~ ., data = airquality, na.action = "na.impute")
# Partial plot of variables.
plot.variable(airq.obj)
```

8.4. 8.4. Classification

There are three split rules that can be used to grow classification trees. In this model, the y-outcome associated with an individual i is a single single categorical value. Let $\mathbf{p} = (p_1, \ldots, p_J)$ be the class proportions for the classes 1 through J respectively, for the y-outcome in the node.

Weighted Gini index splitting

Suppose the proposed split for the root node is of the form $x \le c$ and $x > c$ for a continuous x-variable x, and a split value of c. The impurity of the node is defined as

$$\phi(\mathbf{p}) = \sum_{j=1}^{J} p_j(1 - p_j) = 1 - \sum_{j=1}^{J} p_j^2.$$

The Gini index for a split c on x is

$$\theta(x, c) = \frac{n_l}{n}\phi(\mathbf{p}_l) + \frac{n_r}{n}\phi(\mathbf{p}_r),$$

where, as before, the subscripts l and r indicate left and right daughter node membership respectively, and n_l and n_r are the number of cases in the daughters such that $(n = n_l + n_r)$. The goal is to find x and c to minimize

$$\theta(x, c) = \frac{n_l}{n}\left(1 - \sum_{j=1}^{J} \left(\frac{n_{j,l}}{n_l}\right)^2\right) + \frac{n_r}{n}\left(1 - \sum_{j=1}^{J} \left(\frac{n_{j,r}}{n_r}\right)^2\right), \tag{21}$$

where $n_{j,l}$ and $n_{j,r}$ are the number of cases of class j in the left and right daughter, respectively, such that $(n_j = n_{j,l} + n_{j,r})$. This is equivalent to maximizing

$$\theta^*(x,c) = \frac{1}{n}\sum_{j=1}^{J}\frac{n_{j,l}^2}{n_l} + \frac{1}{n}\sum_{j=1}^{J}\frac{(n_j - n_{j,l})^2}{n - n_l}, \tag{22}$$

Unweighted Gini index splitting

Unweighted Gini index splitting corresponds to

$$\theta(x,c) = \phi(\mathbf{p}_l) + \phi(\mathbf{p}_r).$$

The best split is found by minimizing

$$\theta(x,c) = \left(1 - \sum_{j=1}^{J}\left(\frac{n_{j,l}}{n_l}\right)^2\right) + \left(1 - \sum_{j=1}^{J}\left(\frac{n_{j,r}}{n_r}\right)^2\right).$$

This is equivalent to maximizing

$$\theta^*(x,c) = \sum_{j=1}^{J}\left(\frac{n_{j,l}}{n_l}\right)^2 + \sum_{j=1}^{J}\left(\frac{n_j - n_{j,l}}{n - n_l}\right)^2. \tag{23}$$

Heavy weighted Gini index splitting

Heavy weighted Gini index splitting corresponds to

$$\theta(x,c) = \frac{n_l^2}{n^2}\phi(\mathbf{p}_l) + \frac{n_r^2}{n^2}\phi(\mathbf{p}_r).$$

The best split is found by minimizing

$$\theta(x,c) = \frac{n_l^2}{n^2}\left(1 - \sum_{j=1}^{J}\left(\frac{n_{j,l}}{n_l}\right)^2\right) + \frac{n_r^2}{n^2}\left(1 - \sum_{j=1}^{J}\left(\frac{n_{j,r}}{n_r}\right)^2\right).$$

This is equivalent to maximizing

$$\theta^*(x,c) = \frac{1}{n^2}\sum_{j=1}^{J} n_{j,l}^2 + \frac{1}{n^2}\sum_{j=1}^{J}(n_j - n_{j,l})^2 - \frac{n_l^2}{n^2} - \frac{n_r^2}{n^2} + 2. \tag{24}$$

Note that the above is non-negative due to the addition of the constant last term.

Terminal node estimators

The predicted value for a terminal node h are the class proportions in the node. Let individual i have feature $\mathbf{X}_i$. and reside in terminal node h. Let n_j equal the number of bootstrap cases of class j in the node. Then the proportion for the jth class is given by

$$\hat{p_{j,h}} = \frac{1}{n_h}\sum_{\mathbf{X}_i \in h} I\{Y_i = j\}. \tag{25}$$

To produce the OOB predicted value for individual i this is averaged over all `ntree` trees. Let $\hat{p_{j,b}}(\mathbf{X}_i)$ denote the predicted value for the jth proportion for tree $b \in \{1, \ldots, \texttt{ntree}\}$. As before, $I_{i,b} = 1$ if individual i is an OOB individual for $b \in \{1, \ldots, \texttt{tree}\}$, otherwise set $I_{i,b} = 0$. The OOB predicted value for the jth proportion for individual i is

$$p^*_{e,j}(\mathbf{X}_i) = \frac{\sum_{b=1}^{\texttt{ntree}} I_{i,b}\hat{p_{j,b}}(\mathbf{X}_i)}{\sum_{b=1}^{\texttt{tree}} I_{i,b}}.$$

Prediction error

Consider $\mathbf{p}^*_e(\mathbf{X}_i) = (p^*_{e,1}(\mathbf{X}_i), \ldots, p^*_{e,J}(\mathbf{X}_i))$. Let

$$p^*_{e,max}(\mathbf{X}_i) = \max_{1 \le j \le J}(p^*_{e,j}(\mathbf{X}_i)),$$

and let N_j^* equal the number of individuals with class equal to j in the data set. The estimated conditional misclassification rate is given by

$$Err_j = \frac{1}{N_j^*} \sum_{i:Y_i=j} (Y_i \neq p^*_{e,max}(\mathbf{X}_i)). \tag{26}$$

The estimated over all misclassification rate is given by

$$Err = \frac{1}{N} \sum_{i=1}^{N} (Y_i \neq p^*_{e,max}(\mathbf{X}_i)). \tag{27}$$

An example of a classification forest follows:

```
# Edgar Anderson's iris data with morphologic variation of three
# related species.
iris.obj <- rfsrc(Species ~., data = iris, ntree=100)
## Plot the results.
plot(iris.obj)
```

8.5. 8.5. Multivariate

In the multivariate and mixed model, the y-outcomes associated with an individual i can be denoted by $\mathbf{Y}_i = ((Y_{i,1}, \ldots, Y_{i,r})$ where $Y_{i,j}$ is real or categorical for each $j \in \{1, \ldots, r\}$. When $Y_{i,j}$ is real for all $j \in \{1, \ldots, r\}$ the model is a multivariate regression forest. In this case the split rule statistic is a composite of r split rule statistics based on [Weighted Variance Splitting]. The outcomes in the node are normalized to ensure that a particular j-specific statistic does not overwhelm the composite statistic. When $Y_{i,j}$ is categorical for all $j \in \{1, \ldots, r\}$ the model is a multivariate classification forest. In this case the split rule statistic is a composite of r split rule statistics based on [Weighted Gini Index Splitting]. No normalization is necessary since the j-specific statistics represent an impurity measure which is invariant with respect to scale. In the general case where $Y_{i,j}$ is real or categorical for each $j \in \{1, \ldots, r\}$ the model is a multivariate mixed forest. In this case the split rule statistic is a composite of r split rule statistics based on [Weighted Variance Splitting] and [Weighted Gini Index Splitting]. Only those j-specific statistics representing real outcomes are normalized.

Terminal node estimators

There are j sets of terminal node estimators specific to the real or categorical cases previously described.

Prediction error

There is no prediction error implemented in this scenario.

8.6. 8.6. Unsupervised

Unsupervised models are appropriate in settings where there is no y-outcome. In the unsupervised split rule, a subset of the x-variables (the size of which is specified by the formula) is chosen as the pseudo y-outcomes at each node, and the multivariate split rule in the [Multivariate] section is applied. Thus, each `mtry` attempt results in a multivariate mixed split rule. Note importantly that all terminal node statistics and error rates are turned off under unsupervised splitting.

Terminal node estimators

There are no terminal node estimators implemented in this scenario.

Prediction error

There is no prediction error implemented in this scenario.

9. 9. Supplementary Code

Generalized code for simulating the spheres used in [Tree Decision Boundary] and elsewhere:

```
```

Generalized code for simulating the survival data set used in [Benchmark of Hybrid Parallel Processing]:

```
```

The primary harness used in [Benchmark of Hybrid Parallel Processinge]:

```
```

The replica harness used in [Benchmark of Hybrid Parallel Processinge]:

10. 10. References
