# Supplementary figures and images for: Combining single‐cell sequencing to identify key immune genes and construct the prognostic evaluation model for colon cancer patients
Source: Clin Transl Med. 2021 Jul 19;11(7):e465. doi: 10.1002/ctm2.465 (PMC8288000; doi:10.1002/ctm2.465)

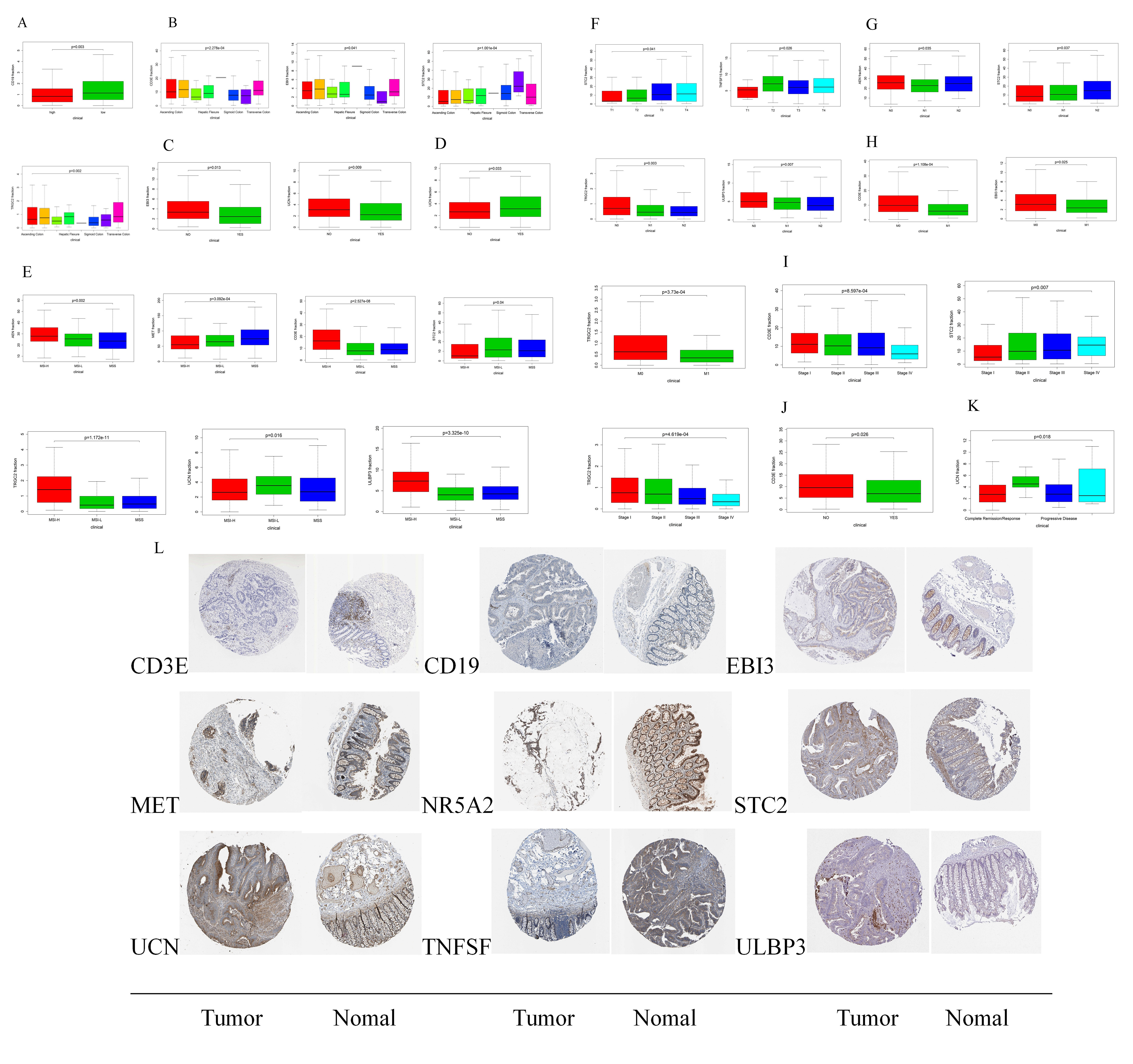

Supplement: Supplementary file 6 — Supporting information. Figure S1 (A) Correlation analysis between the expression of model gene CD19 and patient age. (B) Correlation analysis between model gene expression and tumor anatomical location. (C) Correlation analysis between model gene expression and occurrence of mismatch repair. (D ) Correlation analysis between the expression of model gene UCN and the occurrence of lymphatic invasion. (E) Correlation analysis between the expression of model gene and the occurrence of microsatellite instability. (F) Correlation analysis between model gene expression and tumor TNM staging. (G) Correlation analysis between model gene expression and lymphatic metastasis. (H) Correlation analysis between model gene expression and distant metastasis. (I) Correlation analysis between model gene expression and clinical stage of patients. (J) Correlation analysis between CD3E gene expression and tumor recurrence after treatment. (K) Correlation analysis between UCN gene expression and initial treatment effect. (L) The expression of prognostic‐related immune differential genes in colon cancer tumor tissues and normal tissues [file CTM2-11-e465-s006.jpg]
